# Formation of Temporary Negative Ions and Their Subsequent Fragmentation upon Electron Attachment to CoQ_0_ and CoQ_0_H_2_

**DOI:** 10.1002/cphc.202100834

**Published:** 2022-02-10

**Authors:** João Ameixa, Eugene Arthur‐Baidoo, João Pereira‐da‐Silva, Júlio C. Ruivo, Márcio T. do N. Varella, Martin K. Beyer, Milan Ončák, Filipe Ferreira da Silva, Stephan Denifl

**Affiliations:** ^1^ Institut für Ionenphysik und Angewandte Physik Leopold-Franzens Universität Innsbruck Technikerstraße 25 6020 Innsbruck Austria; ^2^ Center for Biomolecular Sciences Innsbruck (CMBI) Leopold-Franzens Universität Innsbruck Technikerstraße 25 6020 Innsbruck Austria; ^3^ Centre of Physics and Technological Research Departamento de Física Faculdade de Ciências e Tecnologia Universidade NOVA de Lisboa 2829-516 Caparica Portugal; ^4^ Instituto de Física Universidade de São Paulo Rua do Matão 1731 05508-090 São Paulo Brazil

**Keywords:** electron carrier molecules, ubiquinone, dissociative electron attachment, electron scattering, quantum chemistry

## Abstract

Ubiquinone molecules have a high biological relevance due to their action as electron carriers in the mitochondrial electron transport chain. Here, we studied the dissociative interaction of free electrons with CoQ_0_, the smallest ubiquinone derivative with no isoprenyl units, and its fully reduced form, 2,3‐dimethoxy‐5‐methylhydroquinone (CoQ_0_H_2_), an ubiquinol derivative. The anionic products produced upon dissociative electron attachment (DEA) were detected by quadrupole mass spectrometry and studied theoretically through quantum chemical and electron scattering calculations. Despite the structural similarity of the two studied molecules, remarkably only a few DEA reactions are present for both compounds, such as abstraction of a neutral hydrogen atom or the release of a negatively charged methyl group. While the loss of a neutral methyl group represents the most abundant reaction observed in DEA to CoQ_0_, this pathway is not observed for CoQ_0_H_2_. Instead, the loss of a neutral OH radical from the CoQ_0_H_2_ temporary negative ion is observed as the most abundant reaction channel. Overall, this study gives insights into electron attachment properties of simple derivatives of more complex molecules found in biochemical pathways.

## Graphical abstract

**In the mitochondrial electron transport chain**, ubiquinone (coenzyme Q10, CoQ_10_) is a mobile electron‐carrier molecule, which gets reduced into ubiquinol (CoQ_10_H_2_). Dissociative electron attachment to their simplest derivatives, CoQ_0_ and CoQ_0_H_2_, in the gas phase has been studied both theoretically and experimentally. Remarkably, despite their structural similarity, the dissociation pathways observed show different behaviors.

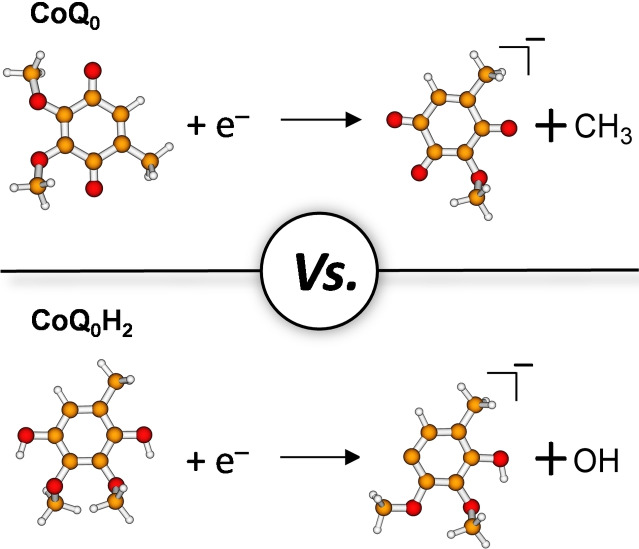

## Introduction

**Figure 1:**
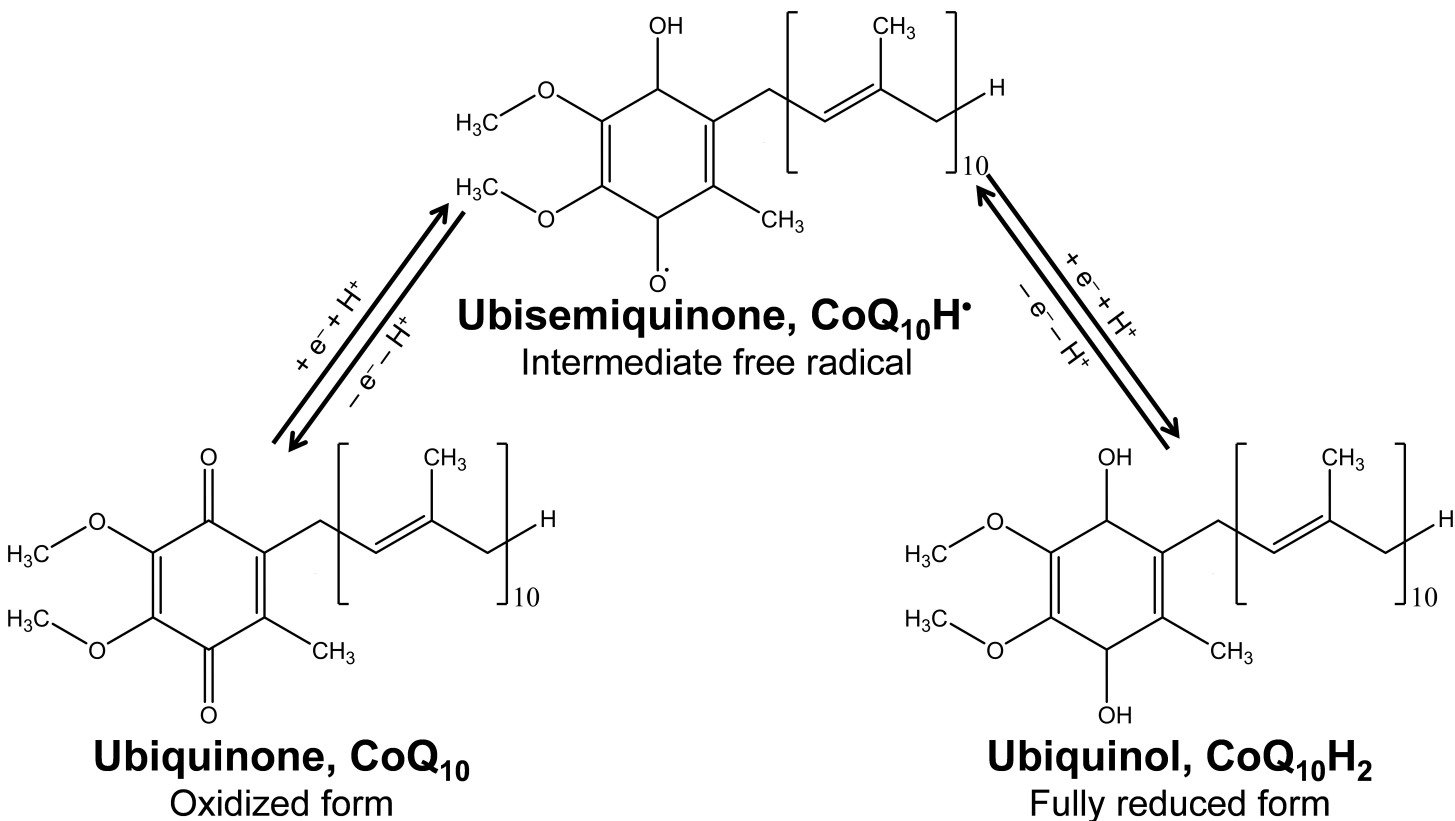
Pathways for the formation of the three forms of the electron carrier ubiquinone, CoQ_10_.

The mitochondria are organelles best known for their role in producing adenosine triphosphate (ATP), that upon conversion to adenosine diphosphate (ADP) releases energy required to power most processes in cells. These organelles, in addition to a chain of four proteins embedded in the inner mitochondrial membrane, identified as complexes I, II, III and IV, make use of mobile molecules – ubiquinone (also called coenzyme Q_10_) and cytochrome *c* – to shuttle electrons down the chain. This electron‐transport chain generates an electrochemical proton gradient across the inner mitochondrial membrane.^[1]^ The enzyme ATP synthase harnesses the energy stored in the electrochemical proton gradient to synthesize ATP. As described by Morton,^[2]^ the simplest electron carrier agent involved in the electron transport chain is coenzyme Q_10_ (CoQ_10_). This molecule consists of a *p*‐benzoquinone (*p*‐BQ, C_6_H_4_O_2_) derivative head‐group accounting for the electron transfer ability, with an attached side group of ten isoprenoid units making this molecule mobile within the inner mitochondrial membrane. In a multi‐step reaction, each complex I and II transfers one electron to a CoQ_10_ molecule reducing it to the intermediate ubisemiquinone radical CoQ_10_H^.^, and then to the fully reduced form known as ubiquinol, CoQ_10_H_2_ (see Figure 1). Subsequently, complex III (cytochrome *bc_1_*) receives a pair of electrons from CoQ_10_H_2_ to regenerate CoQ_10_.^[3]^ In chloroplasts, plastoquinone, a *p*‐BQ derivative, acts as electron carrier in the light‐dependent reactions of photosynthesis.[^4^, ^5^] More recently, the potential use of quinones derivatives for energy harvesting and storage in batteries has been investigated.[^6^, ^7^]

The ability of an electron carrier molecule to attach an electron depends on the availability of a low‐lying vacant molecular orbital.^[8]^ The capture of an electron with kinetic energy below the ionization potential, and thus termed low‐energy electron (LEE), by a target molecule leads to the formation of a temporary negative ion, TNI^#−^, also called resonance. Since TNIs are formed in an electronically or vibrationally excited state (as indicated by the superscript ^#^), they tend to subsequently decay within 10^−15^ up to 10^−2^ s.[^9^, ^10^] The decay of a TNI^#−^ into anionic fragments and one or more neutral counterparts through dissociative electron attachment (DEA) takes place when the time required for dissociation is sufficiently shorter compared to the time for spontaneous emission of the extra electron (autodetachment)^[11]^ which often yields the neutral parent molecule in an excited state.[^12^, ^13^, ^14^] The DEA process was shown to play an important role in radiation damage of biomolecules upon interaction of ionizing radiation with matter, including DNA,^[15]^ proteins^[16]^ as well as many radiosensitizing agents.[^17^, ^18^, ^19^, ^20^] It should be noted, however, that already a small hydration shell changes both energetics and dynamics of the DEA process.[^21^, ^22^]

The lifetime of the TNI^#−^ is conferred by the electron capture mechanism of the target molecule. If the incoming electron electronically excites the target molecule, a core‐excited TNI^#−^ is formed by concomitant capture of the incoming electron in a previously vacant molecular orbital (MO). In the gas‐phase, the lifetime of a core‐excited Feshbach resonance is relatively long with respect to the time for autodetachment, which in turn favors the dissociation of the TNI^#−^, i. e. DEA.^[23]^ Otherwise, a shape resonance is produced when the incoming electron is captured by the target molecule in a potential barrier created by the electron‐molecule interaction. Usually, a TNI^#−^ created by a shape resonance has a lifetime ranging from 10^−15^ up to 10^−10^ s.[^12^, ^14^]

The formation of resonances by electron attachment to *p*‐BQ as well as their lifetimes and decay channels have been studied extensively using a broad range of experimental methods, such as electron attachment or transmission spectroscopy,[^24^, ^25^, ^26^, ^27^, ^28^, ^29^, ^30^, ^31^, ^32^, ^33^] photoelectron spectroscopy,[^34^, ^35^, ^36^] action spectroscopy,[^37^, ^38^, ^39^] as well as theoretical calculations, namely quantum chemical models[^40^, ^41^, ^42^] or electron scattering calculations.[^43^, ^44^, ^45^] Based on time‐resolved photoelectron spectroscopy and *ab initio* calculations, Horke *et al*.^[46]^ demonstrated that the excitation of gas‐phase *p*‐BQ^.−^ anions at 2.58 eV (480 nm) and 3.10 eV (400 nm) yields excited states, namely the shape resonance ^2^A_u_ lying at 0.7 eV and the core‐excited resonance ^2^B_3u_ at 1.35 eV above the ground state of the neutral, that decay eventually on a sub‐40 femtosecond timescale *via* a series of conical intersections to the ground state of the anion, thereby preventing autodetachment. Further, with the same combination of methods, Bull *et al*.^[47]^ showed that a similar mechanism is also operative in CoQ_0_ (2,3‐dimethoxy‐5‐methyl‐*p*‐benzoquinone), an analogue of CoQ_10_ holding no side‐chain. Although *p*‐BQ has an electron affinity of about 1.91 eV,^[48]^ the formation of a long‐lived molecular anion *p*‐BQ^.−^ through stabilization of the core‐excited resonance ^2^B_3u_ at about 1.35 eV was reported by several attachment studies, while no attachment of thermal electrons was observed.[^25^, ^26^, ^33^, ^49^] In addition to the molecular anion *p*‐BQ^.−^, Khvostenko *et al*.^[50]^ identified more than twenty DEA channels. Finally, the recent electron attachment study by Pshenichnyuk *et al*.^[51]^ (using a standard ion source with magnetic mass spectrometer for mass analysis of anions) involving shorter‐tail analogues $\mathrm{Co}Q_{n}$ (*n*=1, 2, 4) revealed that elongation of the side chain leads to an increase of the lifetime of the isolated molecular anions$\;\mathrm{Co}Q_{n}^{•-}$ formed at 1.2 eV as well as to a reduction of the efficiency of dissociative pathways producing fragment anions.

In the present study, we assess the formation of TNIs and their subsequent decay into anionic fragments through the study of the interaction of LEEs with the smallest analogue CoQ_0_ in the gas phase. Anion efficiency curve for fragment anions resulting from the decay of TNI states are determined with a crossed electron‐molecular beam setup coupled with a quadrupole mass spectrometer. Quantum chemical calculations provide thermochemical thresholds for subsequent comparison with the experimentally determined thresholds of the observed DEA reactions. Calculations on elastic electron scattering by CoQ_0_ as well as empirical correction of orbital energies are employed to predict the position of short‐lived TNI states. Moreover, we further studied electron attachment to the reduced analogue of CoQ_0_, the hydroquinone derivative CoQ_0_H_2_ (2,3‐dimethoxy‐5‐methylhydroquinone). A comparison between both CoQ_0_ and *p*‐BQ, CoQ_0_H_2_ and hydroquinone (HQ, C_6_H_4_(OH)_2_) will also be provided, respectively.

## Experimental Section

### Dissociative electron attachment set‐up

The anion efficiency curves for mass‐selected fragment anions formed in electron attachment to CoQ_0_ and CoQ_0_H_2_ were measured with a crossed molecular‐electron beam setup, which consists of a hemispherical electron monochromator (HEM) coupled with a quadrupole mass spectrometer.^[17]^ CoQ_0_ (182 u) and CoQ_0_H_2_ (184 u) sample were purchased from Sigma‐Aldrich and placed as received in an external container kept at 313 K. Given that CoQ_0_/CoQ_0_H_2_ exist in a natural equilibrium, each sample might contain traces quantities of the other. From electron ionization mass spectra at the electron energy of 70 eV carried out before the measurements for anions, we observe a negligible contribution of <0.2 % of CoQ_0_ in the CoQ_0_H_2_ sample at 313 K. The sample vapor is introduced into the interaction region of the HEM by a 1 mm‐diameter stainless steel capillary attached to a gas inlet coupled with a precision valve. Within the interaction region of the HEM, the effusive neutral beam crossed orthogonally with the electron beam. The anions formed therein were extracted by a weak electrostatic field towards the quadrupole mass spectrometer for mass selection. At last, a channel electron multiplier operated in single‐pulse counting mode was employed for ion detection. For a given mass‐selected anion, a complete anion efficiency curve was measured in the electron energy range of 0 to 20 eV as a first step, and subsequent measurements were performed only for the electron energies showing ion yield intensity. The HEM was tuned to generate an electron beam with an energy resolution of 120 meV for transmitted electron currents of 5 up to 30 nA as monitored with a picoammeter connected to a Faraday plate placed after the interaction region. The electron energy resolution was determined by measuring the full‐width at half maximum of the well‐known ∼0 eV resonance for the formation of Cl^−^ from CCl_4_.^[52]^ This reaction was also used to calibrate the electron energy scale of the anion efficiency curves. Finally, the experimental onsets for the observed fragments were determined from the Gaussian fittings of the ion yields, as presented elsewhere.^[53]^ The ion yields presented in this work are shown in arbitrary units (a.u.) and thus ion intensities are not comparable between CoQ_0_ and CoQ_0_H_2_.

### Theoretical Methods

**Figure 2:**
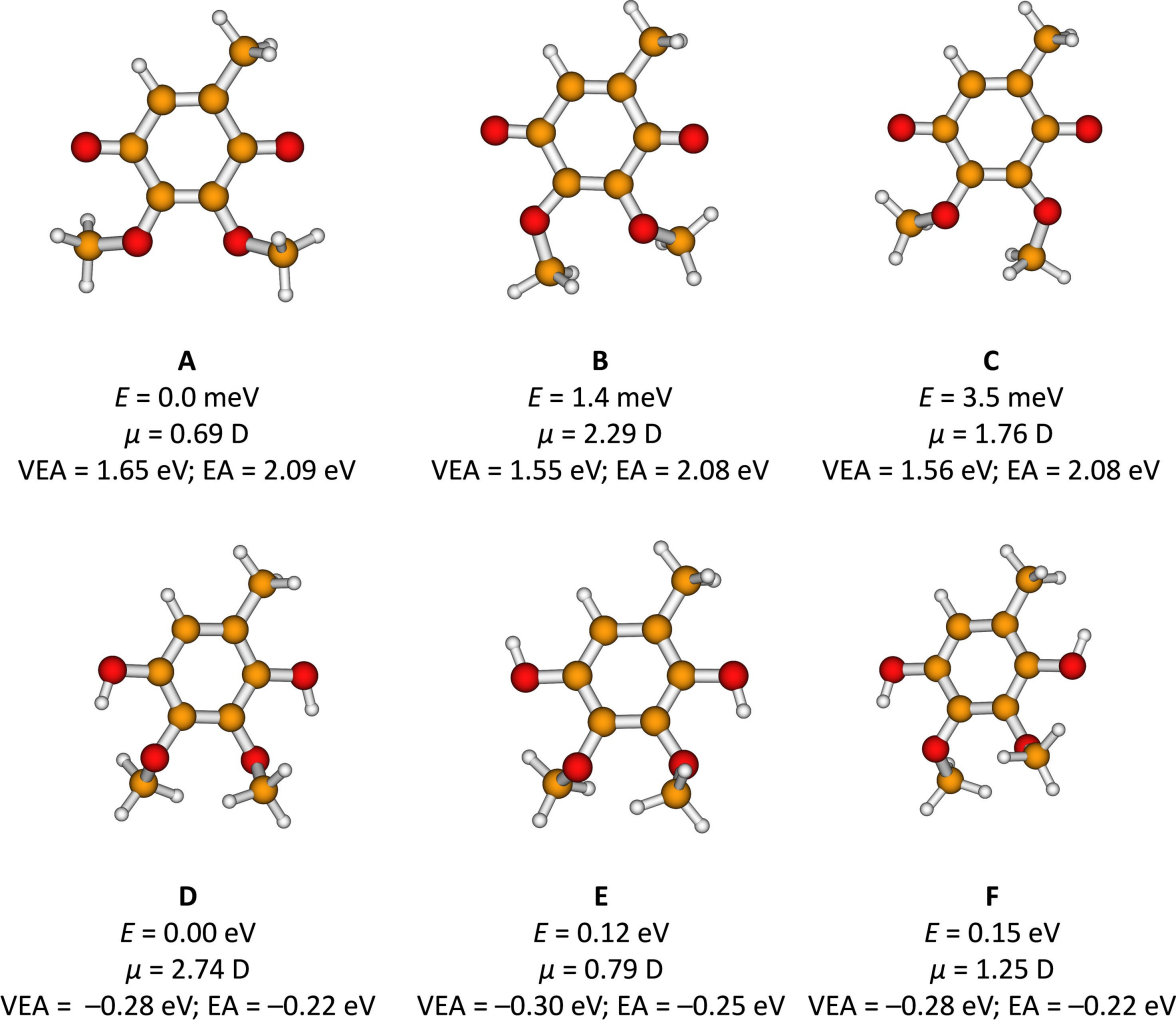
Relative energy *E*, dipole moment *μ*, vertical electron affinity (VEA) and electron affinity (EA) in three optimized isomers of CoQ_0_ (A–C) and CoQ_0_H_2_ (D–F). Calculated at the B3LYP/aug‐cc‐pVDZ level.

Different structures of the CoQ_0_ molecule were searched using the engine built in the Avogadro software^[54]^ and subsequently optimized with density functional theory (DFT), employing the B3LYP functional and the aug‐cc‐pVDZ basis set. We obtained three conformers A–C, see Figure 2, that all lie within 4 meV. The differences in geometry are not expected to significantly impact the π* resonances of interest. The calculated vertical electron affinity (VEA, see below) does not considerably differ among the three conformers (<0.1 eV), suggesting similar spectra of π* shape resonances. The dipole moment of the conformer A is significantly smaller than those of the conformers B and C. We have chosen the most stable A conformer for further calculations as (i) the geometry would not significantly impact the positions of the shape resonances; and (ii) the smaller dipole moment is expected to make the signatures of the anion states more evident in the integral cross section (ICS), in view of the smaller dipolar contribution to the background. For the electron scattering calculations, we utilized the Schwinger Multichannel method^[55]^ implemented with the Bachelet‐Hamann‐Schlüter pseudo‐potentials,^[56]^ SMCPP. The scattering wave function was expanded in a basis of configuration state functions (CSFs), i. e., spin‐adapted (*N*+1)‐electron Slater determinants. In the static‐exchange (SE) approximation, the trial basis set only comprises CSFs of the type $\left|\Phi_{0}\rangle \right|\phi_{j}\rangle$ , where $\Phi_{0}$ is the target ground state, described in the Hartree‐Fock (HF) level, and $\phi_{j}$ is a scattering orbital (the product is properly anti‐symmetrized). Since the target is kept frozen in the SE approximation, the correlation‐polarization effects are not accounted for. In the static‐exchange plus polarization approximation (SEP), the CSF space is augmented with configurations given by $\left|\Phi_{n}\rangle \right|\phi_{j}\rangle$ , where $\Phi_{n}$ is a singly excited target state. While we considered both singlet‐ and triplet‐coupled excitations, only CSFs with total spin S=1/2 were included in the calculation. The SEP configuration space was chosen based on the energy criterion described by Kossoski and Bettega^[57]^ and was composed of 14131 CSFs. Modified virtual orbitals (MVOs) obtained from cationic cores with charge +6 *e* were employed as particle orbitals for the target excitations as well as scattering orbitals. The Gaussian basis sets employed in the target and scattering calculations were the same described in previous study with the *p*‐BQ molecule, see Ref..^[45]^ The target geometry used in the scattering calculations was optimized at the MP2/aug‐cc‐pVDZ level with the Gaussian09 package.^[58]^

The characters of the resonance states of CoQ_0_ were assigned from the inspection of the pseudo‐eigenstates of the scattering Hamiltonian represented in the CSF space. Especially for narrow resonances, there is usually one such pseudo‐state that lies close in energy (<0.2 eV) to the ICS peak related to the resonance, with large coefficients on a few CSFs, typically one 1 to 3, given by products of the target ground state with a MVO. To a reasonable approximation, the orbital occupied by the additional electron to form the shape resonance ($\varphi_{res}$ ) can be written as $\varphi_{res}=\;\Sigma_{i}\;c_{i}\;\chi_{i}$ , where $\chi_{i}$ are the singly occupied VOs of the CSFs of interest, and the coefficients $c_{i}$ are taken from the pseudo‐eigenstate. VEA estimates, computed as empirically corrected virtual orbital energies, were also obtained according to Scheer and Burrow.^[59]^ The geometry optimizations and virtual orbital energy (VOE) calculations were calculated with the B3LYP/6‐31G* model.

The shape and core‐excited resonances of the CoQ_0_ molecule were also computed with the complete active space self‐consistent‐field (CASSCF) method implemented in the OpenMOLCAS package.^[60]^ The dynamic electronic correlation was further accounted for with second‐order perturbation theory (CASPT2). The calculations used the extended relativistic atomic natural orbital (ANO−L) basis set in the [3s2p1d] contraction scheme. The active space comprised the occupied π and *n* orbitals as well as the virtual π* orbitals, amounting to 13 electrons and 10 orbitals, i. e., CASSCF(13,10). The CASPT2 calculations used the state‐average CASSCF wave function as reference, with the imaginary shift^[61]^ set to 0.2 a.u. The ionization‐potential electron‐affinity (IPEA) shift was not employed, as recommended by Zobel *et al*.^[62]^ for organic chromophores.

In Figure 2, selected isomers of CoQ_0_H_2_ are also shown, D−F. Here, energy differences are more pronounced and depend on the orientation of OH groups; VEAs are negative due to the limited basis set. Energies of reaction channels upon electron attachment were calculated at the B3LYP/aug‐cc‐pVDZ level, and the stability of the wave function was tested for all structures. The character of located structures was confirmed through vibrational analysis, relative and reaction energies as well as electron affinities include zero‐point correction; vertical electron attachment energies are reported without the correction. DFT calculations were performed in the Gaussian software package.^[58]^

## Results and Discussion

**Figure 3:**
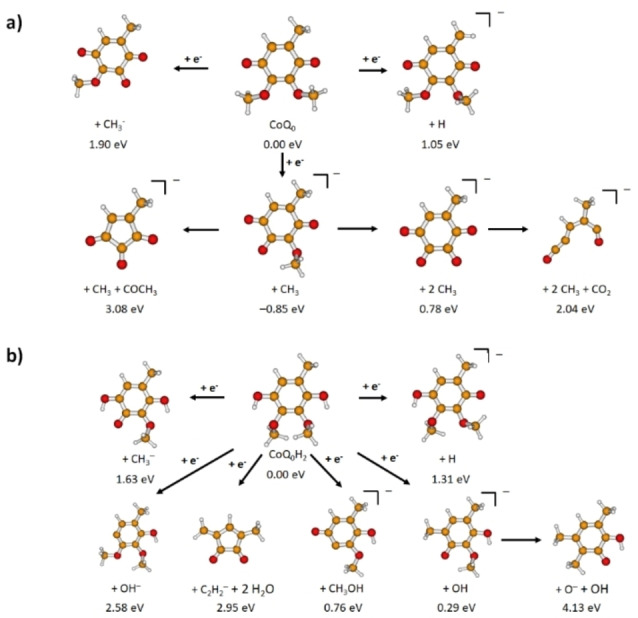
Suggested dissociation pathways in a) CoQ_0_ and b) CoQ_0_H_2_ as calculated at the B3LYP/aug‐cc‐pVDZ level.

Using mass spectrometry, we have identified the anionic species formed by electron attachment to CoQ_0_ and its reduced form, CoQ_0_H_2_. Also, the intact CoQ_0_ molecular anion was observed; however, in the present contribution, we focus on the DEA pathways alone. In contrast, the molecular anion of CoQ_0_H_2_ is not observed in the present experiment. This result is supported by the present calculations that predict a negative electron affinity for CoQ_0_H_2_ (note that the value depends considerably on basis set quality and the negative value may be a computational artefact). In such case, fast autodetachment prevents the experimental detection in a μs timescale. DEA to CoQ_0_ produced six detectable anion fragments through (i) single‐bond ruptures, namely dehydrogenation and single as well as double demethylation reactions, and (ii) rearrangement reactions that yield the fragment anions observed at *m*/*z* 124 and *m*/*z* 108. The observed anions produced in DEA to CoQ_0_ are listed in Table 1, along with the positions of peak maxima observed in the anion efficiency curves, experimental onsets and calculated thermochemical thresholds. It should be noted that the thermochemical thresholds predicted at the B3LYP level consider the initial (neutral molecule) and final states (negative ion fragment and neutral counterpart(s)) of the DEA process. However, rearrangement reactions might involve transition states above the thermochemical threshold which are not considered at the B3LYP level of theory. For CoQ_0_H_2_, we have observed the formation of seven anionic fragments resulting from single‐bond cleavages, namely the dehydrogenated molecular anion, as well as a set of fragments formed due to the release of OH, OH^−^, O^−^, and CH_3_^−^. In addition, more complex reactions involving several bond ruptures and rearrangement reactions yield two fragment anions at *m*/*z* 152 and *m*/*z* 26. Table 2 summarizes the properties of the observed anions upon DEA to CoQ_0_H_2_. Figure 3 shows possible molecular structures of the dissociation products produced due to DEA to both a) CoQ_0_ and b) CoQ_0_H_2_.

**Table 1:** Mass‐to‐charge ratio (*m*/*z*) of the fragment anions formed upon DEA to CoQ_0_, along with the peak positions comprising the ion yields (sorted by increasing energy) as well as the experimental onsets and thermochemical thresholds obtained (zero‐point corrected reaction energies) at the B3LYP/aug‐cc‐pVDZ level of theory.

		Peak position [eV]			Thermochemical threshold [eV]	
m/z	Anion	1.	2.	3.	Exp.	Theory
181	(CoQ_0_−H)^−^	1.8	7.0		1.1	1.05
167	(CoQ_0_−CH_3_)^−^	1.8			0.8	‐0.85
152	(CoQ_0_−2CH_3_)^−^	2.9	3.5	5.2	2.2	0.78
124	${C_{6}H_{4}O}_{3}^{-}$	5.5	6.6		4.6	3.08
108	C_6_H_4_O_2_^−^	5.3	7.0	9.3	3.7	2.04
15	CH_3_^−^	8.1	9.7		6.0	1.90

**Table 2:** Mass‐to‐charge ratio (*m*/*z*) of the fragment anions formed upon DEA to CoQ_0_H_2_, along with the peak positions comprising the ion yields (sorted by increasing energy) as well as the experimental onsets and thermochemical thresholds obtained (zero‐point corrected reaction energies) at the B3LYP/aug‐cc‐pVDZ level of theory.

		Peak position^[a,b]^ [eV]			Thermochemical threshold [eV]	
*m/z*	Anion	1.	2.	3.	Exp.	Theory
183	(CoQ_0_H_2_−H)^−^	1.7	2.4		1.3	1.31
167	(CoQ_0_H_2_−OH)^−^	1.6			0.7	0.29
152	(CoQ_0_H_2_−CH_3_OH)^−^	2.5	3.5	4.7	1.7	0.76
26	C_2_H_2_^−^	2.0	5.9		2.9	2.95
17	OH^−^	7.0	9.3		4.0	2.58
16	O^−^				4.0	4.13
15	CH_3_^−^	9.0			6.3	1.63

[a] The peak at 2.0 eV for the anion with *m/z* 26, C_2_H_2_^−^, can be assigned to an impurity, see text for further details. [b] The exact peak positions for the anion with *m/z* 16, O^−^, are not given due to the relatively weak ion yield intensity.

**Figure 4:**
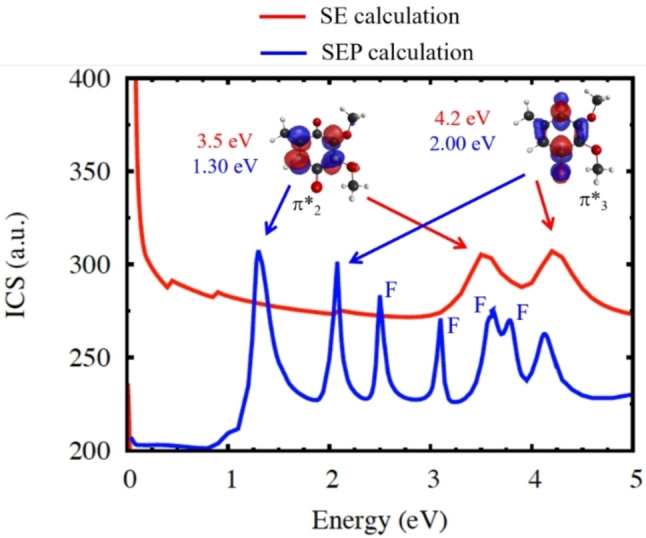
Integral cross section (ICS) for elastic electron scattering by the CoQ_0_ molecule. The red and blue lines correspond, respectively, to the results obtained in the SE and SEP approximations. Virtual orbitals associated with the shape resonances (π_2_* and π_3_*) are also shown. The arrows connect these plots to the SE and SEP level peaks arising from those anion states, and the peak positions in both approximations are indicated alongside the orbital plots. In the SEP cross section, we also assign the peaks around 2.50 eV, 3.08 eV, 3.61 eV and 3.78 eV to Feshbach (F) resonances.

Electron scattering calculations for conformer A of CoQ_0_ are shown in Figure 4. The SE calculations are known to overestimate the energies of shape resonances since the dynamical response of the target electrons to the projectile (correlation‐polarization effects) is neglected. The SE‐level cross section points out three resonances, labelled π_1_* to π_3_* in order of increasing energy, around 0.05 eV, 3.5 eV and 4.2 eV. The SEP results, which incorporate the correlation‐polarization effects, show only two shape resonances, at 1.30 eV (π_2_*) and 2.06 eV (π_3_*). The lowest lying π_1_* anion state is stable and found at 1.9 eV below the neutral form, according to the diagonalization of the scattering Hamiltonian represented in the CSF basis employed in the calculations.

The orbitals in Figure 4 provide insights into the resonance characters of the CoQ_0_ molecule. Although they were obtained with a compact basis set (B3LYP/6‐31G* calculation), their amplitudes are consistent with those of the resonance orbitals, as inferred from the pseudo‐eigenstates of the scattering Hamiltonian. The SEP cross sections in Figure 4 also show several higher‐lying peaks, starting at about 2.5 eV. Since these structures have no counterparts in the SE cross sections, they might be either pseudo‐resonances, which can arise because the open electronic channels are treated as closed in the SEP calculations, or Feshbach resonances. Most of the structures could be assigned as Feshbach resonances employing the same procedure described in Ref.^[45]^ for *p‐*BQ.

However, the description of Feshbach resonances in SEP calculations is generally poor. The resonance positions are significantly overestimated since the parent states are described by single excitations. In view of these limitations, we also performed CASSCF/CASPT2 calculations for the CoQ_0_ anion isomer. To this end, we considered the geometry of the neutral isomer A optimized with second order Møller‐Plesset perturbation theory (MP2). The results are shown in Table 3, along with the SEP results. The Feshbach resonance positions obtained in the SEP calculations are largely overestimated with respect to the CASPT2 excitation energies, as expected, and some states described by the latter method are not present in the SEP‐level cross section. The CASPT2 results also indicate a resonance with mixed character at 1.15 eV, (π_3_*)^1^/(π_3_*)^1^, (π_1_*)^2^, which corresponds to two resonances with predominant shape (2.06 eV) and Feshbach (3.08 eV) characters in the SEP computations. The lowest‐lying triplet states at the CASPT2 level lie around 2.58 eV to 2.81 eV, so the CASPT2 anion states around and above 3.5 eV have core‐excited shape character.

**Table 3:** Energies of the CoQ_0_ anion states for conformer A, in units of eV, obtained from the CASSCF/CASPT2//MP2/aug‐cc‐pVDZ calculations. For each state, the main electronic configurations, and the respective weights (squared coefficients) are indicated. Whenever possible, the resonance positions obtained from SEP‐level scattering calculations are also shown. The state with mixed character, (π_3_*)^1^/(π_3_*)^1^, (π_1_*)^2^, shows up as two resonances with prevailing shape (S) and Feshbach (F) character in the SEP results.

Anion state	weight	CASPT2 [eV]	SEP [eV]
(π_2_*)^1^	0.82	0.85	1.30
(*n*_2_)^1^(π_1_*)^2^	0.87	1.11	3.61
(π_3_*)^1^ (π_3_)^1^(π_1_*)^2^	0.31 0.39	1.15	2.06 (S) 3.08 (F)
(*n*_1_)^1^(π_1_*)^2^	0.88	1.16	3.78
(π_4_)^1^(π_1_*)^2^	0.70	1.46	2.50
(π_3_*)^1^ (π_4_)^1^(π_1_*)^1^(π_2_*)^1^	0.35 0.24	1.69	
(*n*_1_)^1^(π_1_*)^1^(π_2_*)^1^	0.57	3.58	
(*n*_2_)^1^(π_1_*)^1^(π_2_*)^1^	0.59	3.61	
(π_2_)^1^(π_1_*)^2^	0.56	3.64

The unusually large number of low‐lying Feshbach resonances found in CoQ_0_ should allow for the coupling among the shape and core‐excited anion states. Although one cannot obtain resonance widths for the CASSCF/CASPT2 calculations, they seem to account for those couplings better than the SEP scattering calculations, which poorly describe the energies of the parent neutral states (single excitations). It should be noted that the rotation of the methoxy groups, which mainly distinguishes three conformers A–C, seems to have a mild impact on the anion state energies. We further investigated the effect of geometry on the energies of the anion states with different theory levels, as shown in Table S1.

**Figure 5:**
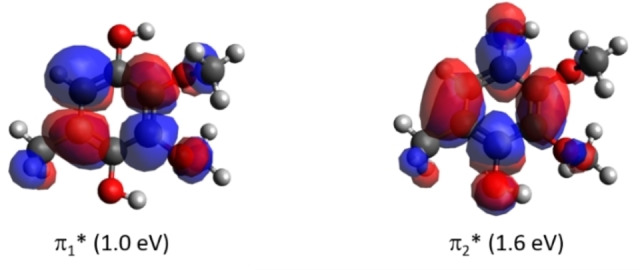
Calculated orbitals relevant to the two lowest lying shape resonances in CoQ_0_H_2_ predicted at the B3LYP/aug‐cc‐pVDZ level.

While we did not perform high‐level calculations for CoQ_0_H_2_, the positions of shape resonances were investigated with empirical VEA estimates based on B3LYP/6‐31G* computations. We obtained two π* shape resonances around 1.0 eV (π_1_*) and 1.6 eV (π_2_*), as shown in Figure 5. The comparison of the empirical VEAs for the shape resonances of CoQ_0_ and CoQ_0_H_2_ is available in Table S2. It should also be mentioned that, unlike CoQ_0_, the CoQ_0_H_2_ molecule has no vertical bound state.

### Dehydrogenation of CoQ_0_ and CoQ_0_H_2_

**Figure 6:**
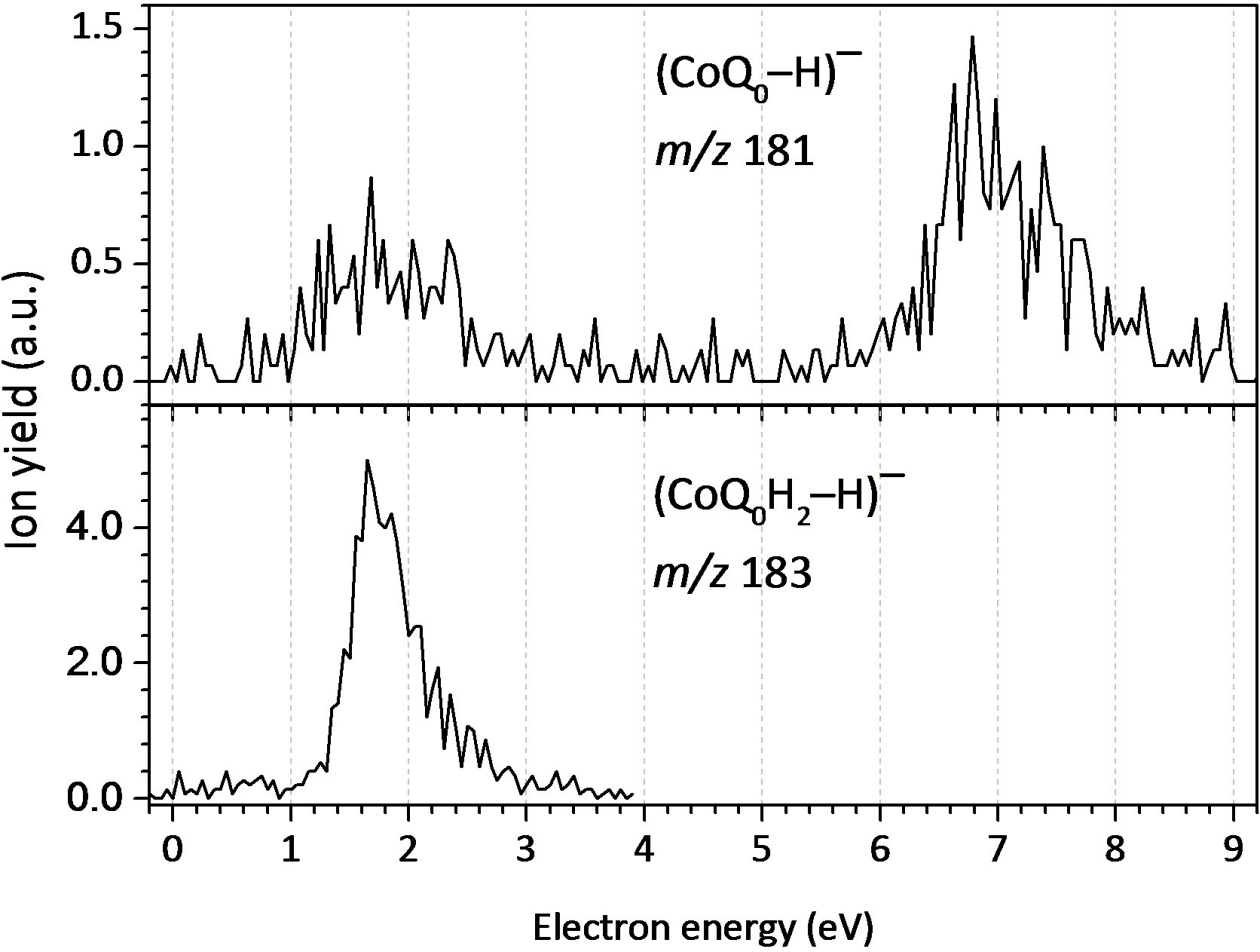
Anion efficiency curve for the dehydrogenated molecular anion formed upon DEA to CoQ_0_ and CoQ_0_H_2_, top panel (CoQ_0_−H)^−^, and bottom panel (CoQ_0_H_2_−H)^−^.

In our DEA study with CoQ_0_, we detected anion yield at *m/z* 181, assigned to the dehydrogenated CoQ_0_ molecular anion, (CoQ_0_−H)^−^. The anion efficiency curve shown in Figure 6 indicates resonances at 1.8 eV and 7.0 eV. The experimental onset of 1.1 eV agrees with the calculated thermochemical threshold of 1.05 eV, assuming H‐abstraction from one of the methyl groups, see Figure 3 including suggested dissociation pathways (H abstraction from the ring or an OCH_3_ group lies higher by more than 0.7 eV). Due to the symmetric shape of the first peak and its position at 1.8 eV, we assign the DEA ion yield rather to the decay of the π_3_* resonance (located at 2.00 eV, see SEP results in Figure 4), without significant contribution of π_2_* at 1.30 eV. The peak at 7.0 eV is above the range of energies considered in the scattering calculations but can be likely assigned to core‐excited resonance. For electron attachment to *p*‐BQ, Khvostenko *et al*.^[50]^ have observed the formation of (*p*‐BQ−H)^−^ through resonances centered at 1.7, 4.86 and 6.46 eV. By comparing the peak positions in the anion efficiency curves for the dehydrogenation of both CoQ_0_ (obtained here) and *p*‐BQ,^[50]^ it can be derived that the dehydrogenation of both quinone analogues occurs at similar electron energies close to 1.8 and 7.0 eV while the core‐excited resonance at 4.86 eV is absent in CoQ_0_. In DEA studies to HQ, Pshenichnyuk *et al*.^[63]^ reported the formation of the dehydrogenated molecular anion of HQ at 1.6 eV as the most intense dissociation pathway, in addition to a weaker contribution at 4.2 eV. Here, we have observed the dehydrogenation of CoQ_0_H_2_ through a single asymmetric contribution comprised of two peaks at 1.7 and 2.4 eV, whereas the resonance at 4.2 eV seems not to be present in the more complex analogue, CoQ_0_H_2_. The loss of an H atom from CoQ_0_H_2_ due to an O−H bond cleavage has a thermochemical threshold of 1.31 eV, i. e., close to the experimental onset. Therefore, we assign the measured yield to the π_2_* shape resonance of CoQ_0_H_2_ (located at 1.6 eV, see Figure 5). We just note that the release of H_2_ or H+H from CoQ_0_ as well as CoQ_0_H_2_ was not detected here while such pathway was observed in electron attachment to HQ^[63]^ but not for *p*‐BQ.^[50]^

### Demethylation of CoQ_0_ and CoQ_0_H_2_

**Figure 7:**
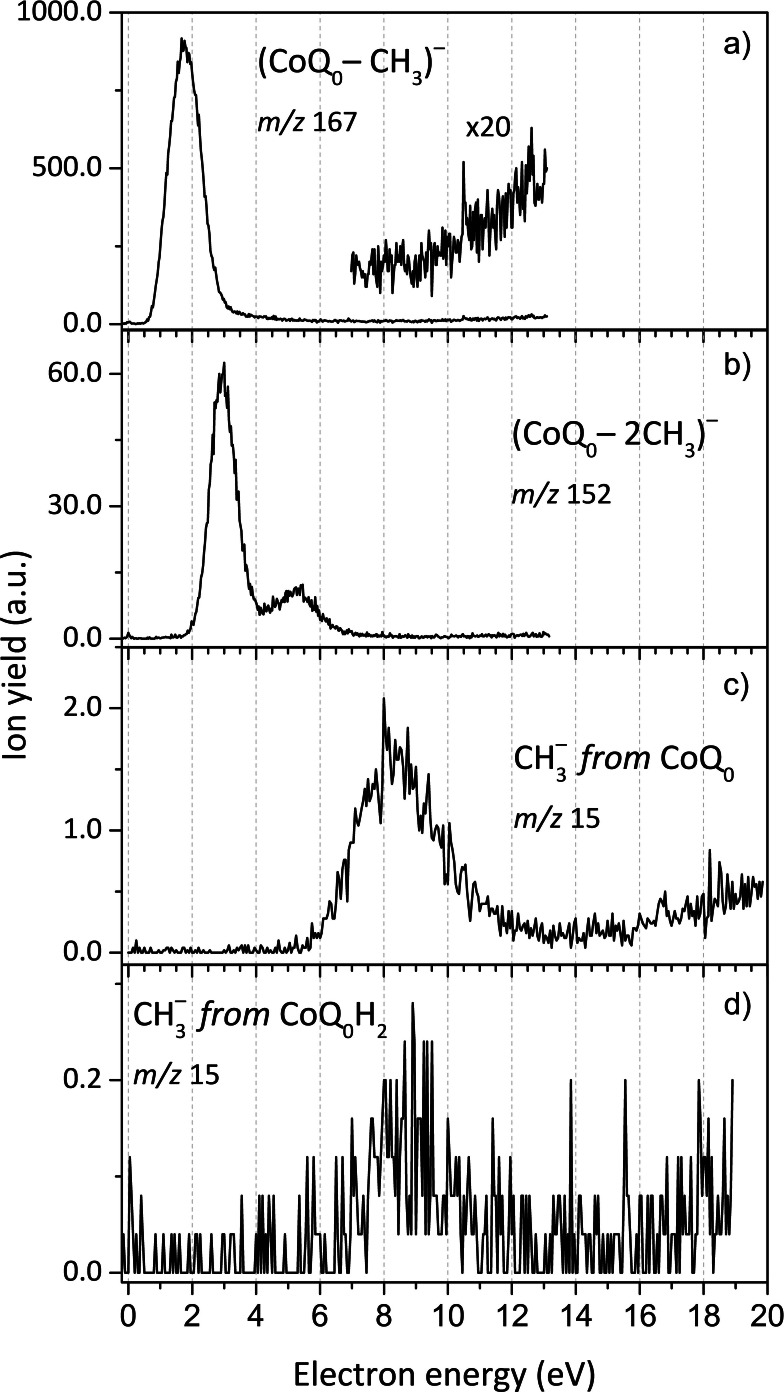
Anion efficiency curves as a function of the electron energy for the formation of the following fragment anions: a) (CoQ_0_−CH_3_)^−^ from CoQ_0_, the inset (x20) shows the signal for ion‐pair formation; b) (CoQ_0_−2CH_3_)^−^; c) CH_3_^−^ from CoQ_0_ and d) CH_3_^−^ from CoQ_0_H_2_.

The demethylation channel resulting in the release of one or two methyl groups is the most intense fragmentation channel observed in CoQ_0_. In Figure 7‐a), the anion efficiency curve for the formation of (CoQ_0_−CH_3_)^−^ shows an intense peak positioned at 1.8 eV arising from DEA, as well as a gradually increasing ion signal for electron energies above ∼9.0 eV which can be assigned to non‐resonant ion‐pair formation. Through the latter process, a fragment negative ion is formed along with a positively charged counterpart ion and an electron. The minor peak at ∼0 eV can be assigned as an artefact or to form upon DEA to thermally excited CoQ_0_. Like for (CoQ_0_−H)^−^, the major peak at 1.8 eV is close to the energies of the π_3_* resonance in CoQ_0_ (2.0 eV). The most probable demethylation pathway proceeds from a methoxy group of CoQ_0_, with the threshold of −0.85 eV. The release of the methyl group directly from the CoQ_0_ ring costs 1.92 eV and might thus also contribute to the tail at higher electron energies. Demethylation in p‐BQ appeared to proceed through a resonance at 1.78 eV, in addition to two further resonances at 5.78 and 6.78 eV.^[50]^ For p‐BQ, Khvostenko *et al*.^[50]^ proposed that the release of a CH_3_ group involves the transfer of a hydrogen atom to a carbon atom followed by a ring opening reaction, while the demethylation of CoQ_0_ upon electron attachment seems to result from a single bond cleavage alone.

DEA to CoQ_0_ also yields a fragment anion at *m*/*z* 152 due to a concurrent loss of two methyl units. The anion efficiency curve shown in Figure 7‐b) is comprised of a very weak ∼0 eV peak (which is assigned to an artefact), and a feature constituted by two peaks appearing at about 2.9 and 3.5 eV. In this energy range, the CASPT2 calculations suggest several Feshbach resonances around 3.5 eV (see Table 3). A broad resonance between 4.0 and 7.0 eV with a maximum at 5.2 eV is also observed. The DEA reaction with the loss of two CH_3_ units due to two O−CH_3_ bond cleavages to form (CoQ_0_−2CH_3_)^−^ is calculated to require 0.78 eV and lies thus energetically below the experimental threshold of 2.2 eV. When the CH_3_ unit from the ring is released along with the second CH_3_ unit from one of the methoxy moieties, the thermochemical threshold rises to 3.17 eV.

As shown in Figure 7‐c), the anion efficiency curve for the formation of the counterpart ${\mathrm{CH}}_{3}^{-}$ from CoQ_0_ exhibits a slightly asymmetric feature possibly comprised of two resonances centered at 8.1 and 9.7 eV, as obtained from a fitting of the ion signal with two Gaussian functions. The onset for ${\mathrm{CH}}_{3}^{-}$ formation due to DEA to CoQ_0_ was experimentally estimated to be 6.0 eV, which is above the respective calculated threshold of 1.90 eV for release from a methoxy moiety. For electron energies above 15.0 eV, ${\mathrm{CH}}_{3}^{-}$ is also produced by a non‐resonant ion‐pair formation process.

For CoQ_0_H_2_, we surprisingly have not observed the release of one or two neutral methyl groups producing demethylated molecular anions. From a computational point of view, the DEA reaction leading to the release of one neutral methyl group would be even exothermic with −0.38 eV. The release of two neutral methyl groups in DEA to CoQ_0_H_2_ would be only slightly endothermic with 0.93 eV, i. e., the non‐observation of these species cannot be explained by thermodynamic arguments. However, ${\mathrm{CH}}_{3}^{-}$ is produced in DEA to CoQ_0_H_2_ through a rather weak resonance centered at 9.0 eV, shown in Figure 7‐d). The experimental onset of 6.3 eV lies substantially above the thermochemical threshold of 1.63 eV for ${\mathrm{CH}}_{3}^{-}$ formation from CoQ_0_H_2_. The electron attachment study by Pshenichnyuk *et al*. did not report the loss of a single methyl unit upon DEA to HQ, which would require a complex DEA reaction with multiple bond cleavage and formation of new bonds.^[63]^

### Loss of OH and O upon DEA to CoQ_0_H_2_

**Figure 8:**
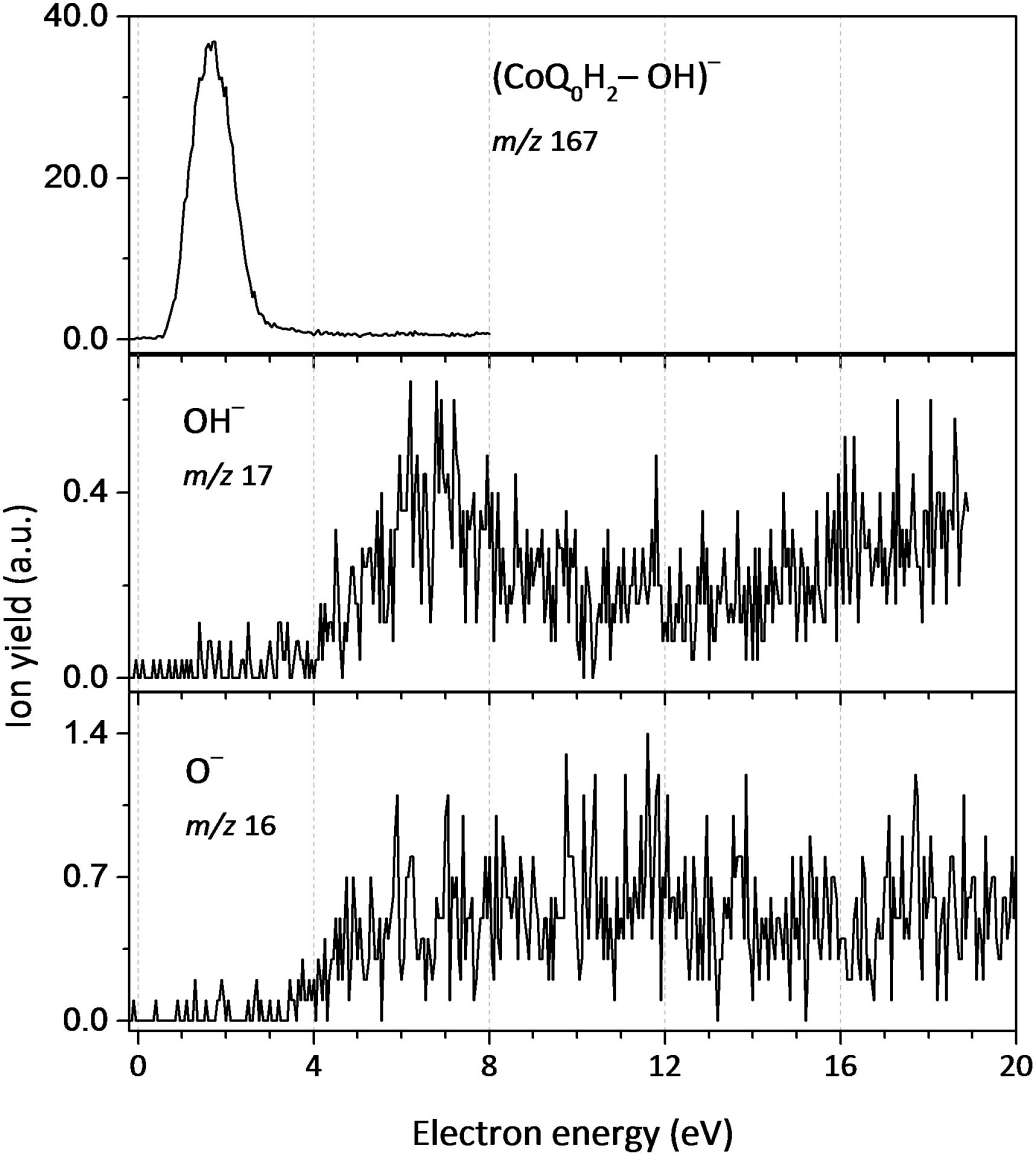
Anion efficiency curve as a function of the electron energy, for the formation of the following fragment anions in DEA to CoQ_0_H_2_: top panel – (CoQ_0_H_2_−OH)^−^, centre panel – OH^−^ and bottom panel – O^−^.

In the present study with CoQ_0_H_2_, the most intense DEA channel is the formation of (CoQ_0_H_2_−OH)^−^ along with loss of a hydroxyl radical through a single peak at 1.6 eV. This energy matches with the suggested π_2_* shape resonance in CoQ_0_H_2_, see Figure 5. The counterpart anion OH^−^ is also detected, and both efficiency curves are shown in Figure 8. Both channels are not detected in DEA to CoQ_0_. OH^−^ formation proceeds through a weak resonance centered at 7.0 eV as well as through an ion‐pair formation pathway at electron energies above 15.0 eV. In previous DEA studies with HQ,^[63]^ OH^−^ was reported to be formed in a single resonance at 10.2 eV, but the loss of neutral OH radical was not observed. Instead, the authors in Ref.^[63]^ observed the formation of (HQ−H_2_O)^−^ with a maximum at 1.3 eV. However, the intensity of this anion was rather weak (0.57 % of the (HQ−H)^−^, which was the most abundant anion observed). At last, anionic oxygen O^−^ was also detected. Note that under the present experimental conditions, the ion yield intensity is relatively small, and thus hindering the identification of resonances in the anion signal observed between 4 and 12 eV. Calculated energy thresholds agree well with the experimental ones (Table 2).

### Other Anions Formed upon DEA to Either CoQ_0_ or CoQ_0_H_2_

**Figure 9:**
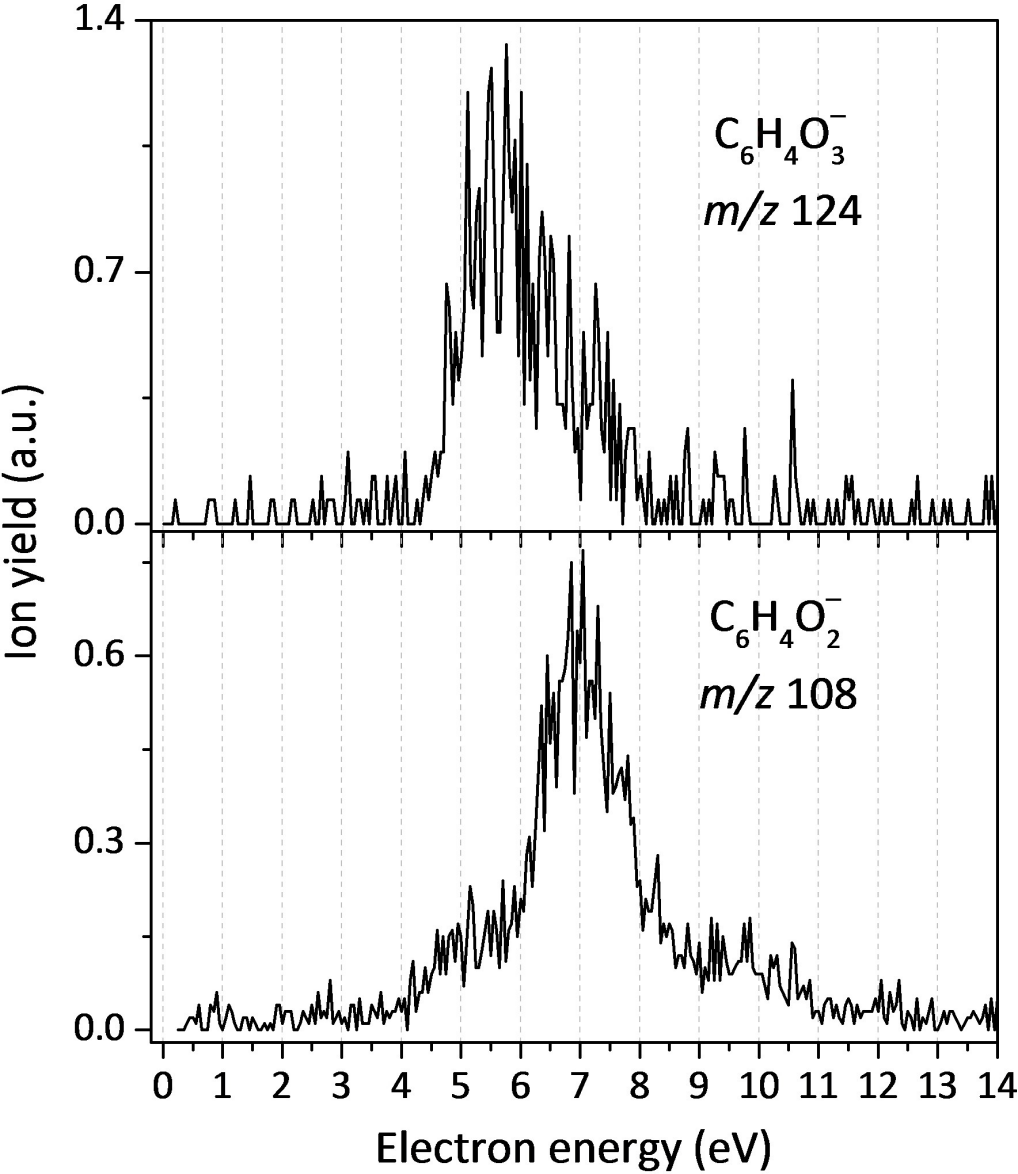
Anion efficiency curves for the formation of the anionic fragments with *m/z* 124, C_5_HO_3_CH_3_^−^(top), and *m/z* 108, C_6_H_4_O_2_^−^ (bottom) from CoQ_0_.

In addition to single‐bond cleavages, DEA to CoQ_0_ also proceeds through more complex reaction pathways, which involve several bond cleavages followed by rearrangements. As shown in Figure 9, we obtained anion yield at *m/z* 124, which we assign to C_6_H_4_O_3_^−^. The formation of this anionic fragment occurs through two resonances at 5.5 and 6.6 eV, with an experimental onset of 4.6 eV. The fragmentation pathway may include the concomitant loss of neutral CH_3_ and COCH_3_ units by multiple bond ruptures within CoQ_0_, namely a cleavage of C−O bond within a methoxy moiety, along with release of the second methoxy moiety accompanied by a carbon atom from the ring. The thermochemical threshold for such dissociation reaction, yielding a five membered ring anion, is 3.08 eV, i. e., this channel might be open at the observed resonance energies. For the ion yield obtained at *m*/*z* 108, which we assign to C_6_H_4_O_2_^−^, the anion efficiency curve shows three different resonances at 5.3, 7.0 and 9.3 eV. Assuming the ring opens upon loss of CO_2_ from the initially formed (CoQ_0_−2CH_3_)^−^ ion, an energy threshold of 2.04 eV is obtained (see Figure 3).

**Figure 10:**
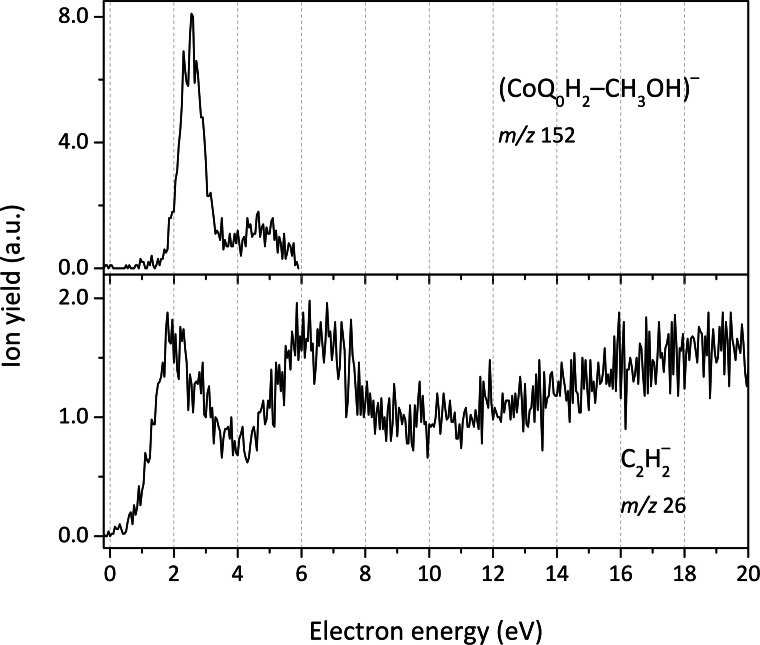
Anion efficiency curves for the formation of anionic fragments at *m/z* 152 (CoQ_0_H_2_−CH_3_OH)^−^ (top panel), and at *m/z* 26 $C_{2}H_{2}^{-}$ (bottom).

In the case of the hydroquinone derivative CoQ_0_H_2_, we detected two additional anionic fragments at *m*/*z* 152 and at *m*/*z* 26 whose anion efficiency curves are presented in Figure 10. The first species, (CoQ_0_H_2_−CH_3_OH)^−^ is most probably formed through intramolecular hydrogen transfer from OH to CH_3_O forming CH_3_OH that leaves the temporary negative ion, with resonances at 2.5, 3.5 and 4.7 eV. The respective calculated reaction energy is 0.76 eV. We also observed weakly abundant anion yield at *m*/*z* 26, which shows anion formation over a broad energy range. In detail, a first asymmetric peak is observed close to 2 eV, which can be most likely ascribed to an impurity.^[64]^ A second resonance is found at 5.9 eV, on top of a non‐resonant ion signal resulting from ion‐pair formation. The fragment anion is assigned to the vinylidene anion $C_{2}H_{2}^{-}$ which has an electron affinity of 0.48 eV.^[65]^ The ring has to decompose during ion formation and a complicated decomposition pathway might be expected, one thermochemically possible scenario takes place through dissociation of two water molecules (Figure 3).

## Conclusion

In the present study, we investigated the formation of temporary negative ions of 2,3‐dimethoxy‐5‐methyl‐p‐benzoquinone (CoQ_0_) and 2,3‐dimethoxy‐5‐methylhydroquinone (CoQ_0_H_2_), as well as their dissociation into fragment anions in the gas phase. Both prototypal molecules are respectively associated with ubiquinone serving as a mobile electron carrier within the mitochondrial electron transport chain, and its fully reduced form, ubiquinol. We observed a variety of DEA channels, however, the presence of the two hydrogen atoms as in CoQ_0_H_2_ substantially alters fragmentation channels. Noteworthy, the most abundant DEA fragment anion of CoQ_0_, (CoQ_0_−CH_3_)^−^, is fully quenched in CoQ_0_H_2_ and, instead, (CoQ_0_H_2_−OH)^−^ is observed as the most intense fragment anion. These fragment anions share a similar resonance peak with its maximum around 1.6–1.8 eV.

The loss of a neutral methyl group was also observed for other derivatives of the coenzyme Q_0_, CoQ_1_, CoQ_2_ and CoQ_4_.^[51]^ Interestingly, the authors in Ref.^[51]^ also observed an anion with a mass 30 u lower than the parent mass, which they assigned to (CoQ_*x*_−OCH_2_)^−^ (*x*=1,2,4), i. e. the abstraction of a formaldehyde molecule. They predicted exothermic reaction energies of about −2.0 eV for this channel, irrespective of the number of the isoprenyl units. Our quantum chemical calculations based on the endothermic release of two single methyl groups are in line with the experimentally obtained threshold of 2.2 eV.

For CoQ_0_, the shape resonance π_3_* predicted at 2.0 eV by the SMCPP scattering calculations decays by DEA reactions leading to loss of a hydrogen atom and a single methyl unit. In CoQ_0_H_2_, the shape resonance π_2_* can decay by the loss of either a hydrogen atom or a hydroxyl group due to single bond cleavages. We did not detect anions formed by the decay of shape resonances π_2_* in CoQ_0_ and π_1_* of CoQ_0_H_2_ with quadrupole mass spectrometry.

When considering the present results and previous ones in Ref.,^[50]^ it also may be concluded that the total number of abundant fragment anions does not seem to increase with the size and the number of the isoprenyl units. This supports the view of the *p*‐BQ unit acting as the electrophore. However, while gas‐phase studies allow to identify TNIs and subsequent dissociation products unambiguously, it is important to further understand how the incorporation of intermolecular interactions, such as within a microsolvated biomolecular cluster, affects the dynamics and energetics of the TNIs of CoQ_0_ and CoQ_0_H_2_ observed in the gas‐phase.

## Conflict of interest

The authors declare no conflict of interest.

1

## Supporting information

As a service to our authors and readers, this journal provides supporting information supplied by the authors. Such materials are peer reviewed and may be re‐organized for online delivery, but are not copy‐edited or typeset. Technical support issues arising from supporting information (other than missing files) should be addressed to the authors.

Supporting Information

## Data Availability

The data that support the findings of this study are available from the corresponding author upon reasonable request.
