# Supplementary material for: Formation of Temporary Negative Ions and Their Subsequent Fragmentation upon Electron Attachment to CoQ0 and CoQ0H2
Source: Chemphyschem. 2022 Feb 10;23(5):e202100834. doi: 10.1002/cphc.202100834 (PMC9306667; doi:10.1002/cphc.202100834)
Supplement: Supplementary file 1 — Supporting Information [file CPHC-23-0-s001.pdf]

ChemPhysChem

Supporting Information

Formation of Temporary Negative Ions and Their Subsequent Fragmentation upon Electron Attachment to CoQ_0 and CoQ_0H_2

João Ameixa,* Eugene Arthur-Baidoo, João Pereira-da-Silva, Júlio C. Ruivo, Márcio T. do N. Varella, Martin K. Beyer, Milan Ončák, Filipe Ferreira da Silva, and Stephan Denifl*

Supporting information to:

1. CoQ_0 anion states

Table S1 summarizes CASSCF/CASPT2 excitation energies of the CoQ0 anion in the neutral molecule structure optimized at different levels. The results obtained with the MP2 geometries for the three conformers are fairly similar, with discrepancies around and below 0.2 eV. The HF geometry for the conformer A gives rise to more significant discrepancies, up to 0.76 eV for the $(\pi_4)^1(\pi_1^*)^2$ state, with respect to the MP2 geometry for the same conformer.

Table S1. Energies of CoQ_0 anion states, in units of eV, obtained for different conformers and geometries optimized for the neutral forms. The geometries of the conformer A were optimized with the HF (A/HF), MP2 (A/MP2) and B3LYP (A/B3LYP) methods, while MP2 as employed for the conformers B and C (B/MP2 and C/MP2). The aug-cc-pVDZ basis set was employed in all optimizations.

**main configuration**	A/MP2 (eV)	A/HF (eV)	A/B3LYP (eV)	B/MP2 (eV)	C/MP2 (eV)
$(\pi_2^*)^1$	0.85	1.19	0.86	1.00	0.96
$(n_2)^1(\pi_1^*)^2$	1.11	1.40	0.67	1.30	1.25
$(\pi_3^*)^1$ $(\pi_3)^1(\pi_1^*)^2$	1.15	1.67	0.86	1.27	1.26
$(n_1)^1(\pi_1^*)^2$	1.16	1.51	1.4	1.39	1.44
$(\pi_4)^1(\pi_1^*)^2$	1.46	2.22	1.65	1.58	1.58
$(\pi_3^*)^1$ $(\pi_4)^1(\pi_1^*)^1(\pi_2^*)^1$	1.69	2.23	1.97	1.80	1.80

2. Comparison of the empirical VAEs for the shape resonances of CoQ_0 and CoQ_0H_2

Table S2. Empirical VAE estimates for the π^* shape resonances (in eV). The results are shown for the A, B and C conformers of CoQ_0 and the D conformer of CoQ_0H_2. The negative energy values indicate bound anion states.

	**CoQ_0**			**CoQ_0H_2**
**Anion state**	A	B	C	D
π_1^*	−1.60	−1.52	−1.53	1.04
π_2^*	0.94	0.99	0.98	1.60
π_3^*	1.37	1.37	1.37	

3. Cartesian coordinates of optimized structures (in Å) and the respective energy including zero-point correction (in Hartree) at the B3LYP/aug-cc-pVDZ level

C2H2-
E = -77.276582
C 0.000000 0.000000 0.857561
C 0.000000 0.000000 -0.487907
H 0.000000 0.927255 -1.108964
H 0.000000 -0.927255 -1.108964

C2H6
E = -79.762099
C -0.255058 0.000000 -0.721417
H 0.577305 0.000000 -1.439954
C 0.255058 -0.000000 0.721417
H -0.577305 -0.000000 1.439954
H 0.871233 -0.887045 0.927817
H 0.871233 0.887044 0.927817
H -0.871233 -0.887044 -0.927817
H -0.871233 0.887045 -0.927817

CH3-
E = -39.818926
C -0.000004 0.000005 0.129804
H -1.028813 0.161004 -0.259615
H 0.374974 -0.971480 -0.259600
H 0.653864 0.810449 -0.259610

CH3
E = -39.814710
C 0.000000 0.000125 -0.000295
H 0.942320 0.543410 0.000590
H -0.942232 0.543561 0.000590
H -0.000087 -1.087718 0.000590

CH3OH
E = -115.691529
C 0.046542 0.667530 0.000000
O 0.046542 -0.759381 0.000000
H -0.441741 1.080395 0.898201
H -0.441741 1.080395 -0.898201
H 1.097372 0.980530 0.000000
H -0.865472 -1.071455 0.000000

CO2
E = -188.602649
O 0.000000 0.000000 1.167317
C 0.000000 0.000000 0.000000
O 0.000000 0.000000 -1.167317

COCH3
E = -153.073993
C -1.075916 0.141313 -0.000131
O 0.323129 -0.435512 0.000125
C 1.329758 0.314515 -0.000074
H -1.558593 -0.245518 -0.901841
H -0.993245 1.232580 -0.004149
H -1.556243 -0.237930 0.906223

H2O
E = -76.423417
O -0.000000 -0.000000 0.117801
H -0.000000 0.764252 -0.471203
H -0.000000 -0.764252 -0.471203

H
E = -0.501657
H 0.000000 0.000000 0.000000

CoQ0H2, D
E = -650.935877
C 0.151740 -0.724750 0.068358
C -1.246504 -0.752709 0.155494
C -1.998773 0.426809 0.042825
C -1.315638 1.631763 -0.160344
C 0.078621 1.675714 -0.226581
C 0.822660 0.496380 -0.091428
O -1.897643 -1.946530 0.346899
C -3.502604 0.368564 0.131608
O 0.712487 2.876543 -0.416632
O 2.202550 0.587715 -0.181347
C 2.900123 0.336735 1.054882
O 0.820961 -1.930143 0.209216
C 1.478293 -2.395878 -0.986331
H -1.869698 2.564269 -0.263005
H -3.826845 -0.041942 1.098931
H -3.937964 1.367986 0.013440
H -3.921470 -0.288367 -0.644373
H 1.935243 -3.356855 -0.726280
H 0.746785 -2.537641 -1.795130
H 2.251160 -1.686016 -1.306710
H 3.965332 0.466704 0.834638
H 2.587547 1.057546 1.824381
H 2.710900 -0.685363 1.406948
H 1.659362 2.687876 -0.499746
H -1.214708 -2.624644 0.460920

CoQ0H2-, D
E = -650.927854
C 0.157493 -0.719430 0.057103
C -1.243544 -0.769557 0.145847
C -2.017495 0.393496 0.048675
C -1.357324 1.619083 -0.137014
C 0.034512 1.687165 -0.197476
C 0.805997 0.520934 -0.072883
O -1.872566 -1.979654 0.324357
C -3.521578 0.300496 0.105850
O 0.651482 2.901757 -0.378757
O 2.174944 0.653075 -0.204300
C 2.946812 0.346523 0.976746
O 0.834613 -1.911342 0.225136
C 1.566498 -2.382293 -0.927752
H -1.928644 2.543345 -0.219547
H -3.860407 -0.155548 1.047675
H -3.975833 1.296106 0.017147
H -3.913199 -0.332906 -0.706531
H 2.010303 -3.341996 -0.633902
H 0.881597 -2.534308 -1.777684
H 2.356536 -1.672535 -1.207156
H 3.995876 0.528619 0.710616
H 2.652972 1.009397 1.806692
H 2.811644 -0.702670 1.271963
H 1.605976 2.724704 -0.420108
H -1.172835 -2.651396 0.384782

CoQ0H2, F
E = -650.930405
C -0.133911 0.723072 0.077279
C 1.261035 0.575465 0.174963
C 1.860428 -0.689696 0.062397
C 1.044031 -1.810022 -0.141894
C -0.340815 -1.681710 -0.222422
C -0.933388 -0.415540 -0.104000
O 1.991849 1.726937 0.380956
C 3.361130 -0.830435 0.166088
O -1.119100 -2.789460 -0.428846
O -2.305932 -0.350226 -0.250573
C -3.034207 0.041005 0.929916
O -0.737550 1.950590 0.218235
C -0.577088 2.836024 -0.901690
H 1.484257 -2.801846 -0.237330

H 3.885200 -0.263663 -0.621264
H 3.742480 -0.480029 1.139859
H 3.659386 -1.880087 0.062588
H -1.132858 3.745671 -0.649478
H 0.481213 3.080274 -1.059893
H -1.001219 2.383493 -1.810264
H -4.093809 0.011452 0.653370
H -2.843884 -0.668417 1.749210
H -2.752795 1.054345 1.238354
H -2.031608 -2.474926 -0.523242
H 2.926213 1.502029 0.456096

CoQ0H2-, F
E = -650.922332
C -0.129548 0.719707 0.081351
C 1.267365 0.580620 0.179467
C 1.869412 -0.685376 0.073721
C 1.051358 -1.807050 -0.131385
C -0.333680 -1.683816 -0.210984
C -0.929756 -0.420206 -0.094847
O 1.982999 1.739287 0.378149
C 3.371768 -0.826587 0.151811
O -1.114510 -2.796448 -0.420163
O -2.302487 -0.358153 -0.262392
C -3.050229 0.037505 0.902553
O -0.739567 1.946763 0.230832
C -0.595080 2.831169 -0.889517
H 1.494976 -2.797546 -0.219062
H 3.887432 -0.226882 -0.621160
H 3.769543 -0.497910 1.125535
H 3.675826 -1.872865 0.006910
H -1.159662 3.737333 -0.635267
H 0.460465 3.084419 -1.056931
H -1.021760 2.374259 -1.796383
H -4.108940 -0.016318 0.618140
H -2.857165 -0.656662 1.736240
H -2.789825 1.060974 1.199725
H -2.028618 -2.477422 -0.495590
H 2.936594 1.561228 0.353411

CoQ0H2, E
E = -650.931307
C -0.028504 0.800718 0.089419
C 1.359977 0.608666 0.156526
C 1.915999 -0.669315 0.023987
C 1.039868 -1.743238 -0.182317
C -0.344750 -1.567253 -0.247719
C -0.898450 -0.285240 -0.095258
O 2.187411 1.682655 0.365964
C 3.409474 -0.856646 0.101150
O -1.220052 -2.611777 -0.454813
O -2.252661 -0.061885 -0.185325
C -3.016110 -0.513136 0.944859
O -0.481194 2.091858 0.285225
C -1.122935 2.701088 -0.852771
H 1.452166 -2.748321 -0.297198
H 3.924707 -0.265751 -0.669871
H 3.801767 -0.517095 1.070772
H 3.679065 -1.911565 -0.032193
H -1.382763 3.718859 -0.541918
H -0.429990 2.738403 -1.706539
H -2.027427 2.146928 -1.127498
H -4.057560 -0.248966 0.731093
H -2.927879 -1.600212 1.068787
H -2.684931 -0.000145 1.860029
H -0.715462 -3.426212 -0.565416
H 1.612858 2.453408 0.494285

CoQ0H2-, E
E = -650.922247
C -0.026472 0.800245 0.083766
C 1.361762 0.607842 0.150333
C 1.914473 -0.671357 0.028302
C 1.040814 -1.750405 -0.171490
C -0.345694 -1.576117 -0.241809
C -0.894577 -0.289654 -0.093659
O 2.191501 1.686868 0.357494
C 3.409165 -0.860743 0.093996
O -1.222981 -2.615558 -0.436348
O -2.249884 -0.062295 -0.199418
C -3.026191 -0.507727 0.920682
O -0.480705 2.090220 0.297461
C -1.117606 2.717231 -0.832439
H 1.448758 -2.756991 -0.266651
H 3.918098 -0.286638 -0.696602
H 3.815729 -0.504337 1.052183
H 3.672888 -1.919809 -0.024046
H -1.374377 3.734746 -0.511273
H -0.422078 2.765031 -1.685772
H -2.023970 2.169294 -1.117595
H -4.065199 -0.231544 0.699219
H -2.949145 -1.596353 1.044585
H -2.699502 0.001100 1.841682
H -0.730426 -3.443151 -0.576461
H 1.611727 2.458878 0.461130

[CoQ0H2-2CH3]-
E = -571.272217
C 1.324596 0.502874 0.000116
C 0.045683 1.234226 -0.000304
C -1.171747 0.440256 -0.000243
C -1.192475 -0.934360 -0.000246
C 0.063835 -1.638168 -0.000167
C 1.251292 -0.951829 0.000068
O -0.068586 2.500280 0.000026
O -2.328102 1.190132 -0.000207
C -2.501162 -1.686537 0.000129
O 2.476486 -1.573014 0.000134
O 2.475863 1.041890 0.000497
H 0.068788 -2.729746 -0.000319
H -3.117130 -1.430516 -0.877268
H -2.328818 -2.772048 -0.008182
H -3.109695 -1.443384 0.886490
H 3.086831 -0.801791 -0.000143
H -1.965402 2.104407 -0.000293

[CoQ0H2-C2H2-2H2O]-
E = -420.704026
C 0.892207 0.746660 -0.000017
C -0.664852 0.825265 0.000007
C -1.146106 -0.578732 -0.000002
C -0.071573 -1.417073 -0.000022
C 1.216258 -0.719295 -0.000020
O -1.326331 1.841987 0.000032
C -2.596942 -0.926723 0.000000
C 2.457168 -1.233798 0.000030
O 1.652928 1.684501 -0.000029
H 2.633701 -2.309632 0.000079
H 3.323275 -0.572290 0.000092
H -0.145019 -2.506364 -0.000045
H -3.095531 -0.494325 -0.879965
H -2.750665 -2.012624 -0.000106
H -3.095493 -0.494490 0.880064

[CoQ0H2-CH3]-
E = -611.135331
C -0.978733 0.788880 -0.139309
C -0.570364 -0.569328 -0.178581

C 0.782661 -0.946414 -0.089938
C 1.807110 -0.010228 0.051698
C 1.427551 1.354682 0.112396
C 0.098412 1.729739 0.028160
O -1.475285 -1.624072 -0.379962
C -2.557827 -1.651121 0.555046
O 1.096105 -2.302070 -0.157619
C 3.249426 -0.446266 0.138058
O -0.320963 3.041846 0.069621
O -2.188339 1.246691 -0.233851
H 2.194546 2.125072 0.222923
H 3.576045 -0.993902 -0.762658
H 3.906076 0.428030 0.255539
H 3.432309 -1.121330 0.991717
H -3.213877 -2.478173 0.248650
H -2.181862 -1.840654 1.576180
H -3.097671 -0.695354 0.530201
H -1.296049 2.917889 -0.035066
H 0.238934 -2.740408 -0.278171

[CoQ0H2-CH3]
E = -611.057147
C -0.880571 0.872678 -0.110487
C -0.642746 -0.549555 -0.125661
C 0.653174 -1.032951 -0.065871
C 1.791642 -0.172342 0.046482
C 1.596563 1.212128 0.094233
C 0.308846 1.728522 0.023204
O -1.625481 -1.489172 -0.303543
C -2.855364 -1.332116 0.436014
O 0.888841 -2.374652 -0.123577
C 3.161126 -0.783507 0.113863
O 0.059477 3.042720 0.054227
O -2.000105 1.440940 -0.207735
H 2.445309 1.888679 0.180793
H 3.367671 -1.382199 -0.785611
H 3.930567 -0.009230 0.208976
H 3.242407 -1.472710 0.967331
H -3.415734 -2.257031 0.265580
H -2.639973 -1.219537 1.508119
H -3.415698 -0.464861 0.075079
H -0.917437 3.110310 -0.035312
H 0.025006 -2.809246 -0.210593

[CoQ0H2-CH3OH]-
E = -535.216255
C 0.739839 1.348825 0.000248
C 0.883955 -0.015710 0.000025
C -0.156417 -0.976853 -0.000049
C -1.511738 -0.569291 0.000088
C -1.748887 0.792830 0.000226
C -0.662573 1.779883 0.000147
O 2.150258 -0.677034 -0.000300
C 3.286573 0.173976 0.000163
O 0.137113 -2.322834 -0.000126
C -2.613489 -1.601176 0.000028
H -2.774719 1.174124 0.000218
H -2.554625 -2.259075 0.881823
H -3.595450 -1.109340 0.000751
H -2.555490 -2.258085 -0.882574
H 4.159620 -0.494625 -0.000176
H 3.293897 0.827736 -0.884413
H 3.293894 0.826776 0.885451
H 1.108329 -2.356357 0.000000
O -0.997251 3.006611 -0.000367

[CoQ0H2-H]-
E = -650.386208
C -0.932922 -0.366692 -0.134474
C -0.086699 0.737119 0.040218
C 1.342967 0.588017 0.181536
C 1.828110 -0.768521 0.067860
C 0.961211 -1.851211 -0.121664
C -0.417634 -1.665223 -0.214530
O -0.688191 1.987889 0.152226
C -0.203248 2.958154 -0.778142
O 2.128199 1.584240 0.387186
C 3.315409 -0.972481 0.192687
O -1.277973 -2.751856 -0.405876
O -2.322788 -0.236665 -0.289966
C -3.003792 0.187492 0.891877
H 1.356901 -2.867323 -0.194050
H 3.861026 -0.396708 -0.573066
H 3.687381 -0.598029 1.160757
H 3.581819 -2.036236 0.096141
H -0.701171 3.904783 -0.523892
H 0.886796 3.054592 -0.686680
H -0.478395 2.670409 -1.808995
H -4.075233 0.206513 0.646925
H -2.829626 -0.521629 1.718837
H -2.671455 1.189582 1.193079
H -2.152420 -2.354745 -0.529831

[CoQ0H2-OH]-
E = -575.184597
C 0.279519 1.715297 0.022944
C -0.902371 0.895185 -0.145265
C -0.636269 -0.518786 -0.173765
C 0.649789 -1.056343 -0.088239
C 1.786152 -0.247340 0.050968
C 1.547544 1.139567 0.106287
O -2.079959 1.388349 -0.257643
O -1.657572 -1.462041 -0.379258
C -2.724682 -1.389298 0.571304
O 0.801456 -2.436004 -0.157726
C 3.167960 -0.846346 0.133762
H 2.413227 1.802677 0.217287
H 3.430434 -1.426946 -0.767896
H 3.919626 -0.051508 0.250151
H 3.277444 -1.538964 0.986476
H -3.471179 -2.133727 0.258929
H -2.358749 -1.641272 1.583112
H -3.152651 -0.378343 0.567371
H -0.103655 -2.766372 -0.279907
C 0.080546 3.208563 0.071094
H 1.042366 3.732283 0.185851
H -0.415600 3.576692 -0.842427
H -0.581789 3.500057 0.903523

[CoQ0H2-OH-O]
E = -499.904889
C -1.335548 -0.891162 0.000006
C -0.126052 -1.592306 0.000035
C 1.162036 -0.975649 0.000047
C 1.272179 0.406412 0.000025
C 0.054722 1.197681 0.000015
C -1.234473 0.499873 0.000016
C 2.395297 -1.847915 -0.000015
O 0.017788 2.457545 0.000014
C 2.595302 1.120990 -0.000034
O -2.318386 1.287017 -0.000008
C -2.679760 -1.572524 -0.000034
H -0.166769 -2.683386 -0.000035
H -3.269947 -1.285563 -0.882181
H -2.564287 -2.663182 -0.000384
H -3.269703 -1.286129 0.882466
H 3.194759 0.852726 0.883499
H 3.194343 0.853292 -0.884035

H 2.441812 2.204682 0.000319
H -1.955214 2.200781 -0.000035
H 2.131462 -2.912368 -0.000324
H 3.023211 -1.654650 -0.882757
H 3.022901 -1.655100 0.883055

[CoQ0H2-OH]
E = -575.032594
C 0.977906 -0.561603 -0.063339
C 0.177057 0.597197 -0.167893
C -1.222508 0.493728 -0.115929
C -1.881857 -0.734957 0.066795
C -1.079693 -1.882697 0.196943
C 0.290254 -1.746491 0.128396
O 0.691985 1.855574 -0.418265
C 1.534584 2.405690 0.611960
O -1.977989 1.626301 -0.256230
C -3.387410 -0.790496 0.122099
O 2.331791 -0.435403 -0.167585
C 3.068259 -1.658723 -0.098284
H -1.546433 -2.856860 0.349175
H -3.833663 -0.409779 -0.808228
H -3.731221 -1.820764 0.275339
H -3.780460 -0.167764 0.938945
H 1.768414 3.427564 0.294460
H 0.999678 2.426817 1.572971
H 2.457537 1.822629 0.709135
H 4.121673 -1.382654 -0.205938
H 2.906359 -2.157938 0.868724
H 2.772386 -2.336777 -0.912232
H -1.360113 2.353857 -0.430205

[CoQ0H2-CO2-2CH3]-
E = -381.413326
O -0.938923 2.099303 -0.000133
C -1.644932 1.115130 0.000127
C -1.377912 -0.293820 -0.000024
C -0.080148 -0.821676 -0.000186
C 1.147651 -0.188256 -0.000540
C 2.417500 -0.213472 0.000046
C -2.579514 -1.219196 0.000212
H -3.221889 -1.068496 0.884477
H -3.222584 -1.067888 -0.883435
H -2.257197 -2.271422 -0.000277
H -0.059132 -1.926833 -0.000143
O 3.622039 -0.091506 0.000329

[CoQ0H2-CH3-COCH3]-
E = -456.718283
C 0.527444 0.849564 -0.000052
C 1.121803 -0.553005 0.000032
C 0.138740 -1.477078 0.000029
C -1.195650 -0.776247 -0.000020
C -0.929120 0.706209 0.000050
O -2.280215 -1.357549 -0.000090
O -1.808677 1.610698 0.000111
O 1.240420 1.874955 -0.000108
C 2.600156 -0.762276 0.000044
H 3.053775 -0.276658 -0.877706
H 3.053773 -0.276569 0.877744
H 2.858948 -1.830379 0.000096
H 0.241041 -2.564228 0.000064

CoQ0, A
E = -649.720248
C -1.434156 0.219215 -0.153800
C -1.561930 -1.272567 -0.018977
C -0.446054 -2.011684 0.112004
C 0.908176 -1.414983 0.174762
C 1.038395 0.062558 0.026070
C -0.088582 0.841052 -0.056006
C -2.948375 -1.840011 -0.062075
O 1.885408 -2.128586 0.385218
O 2.233888 0.678973 0.089665
C 3.426679 0.055364 -0.429327
O 0.063247 2.177134 -0.190970
C -0.836666 3.077576 0.486476
O -2.432541 0.897884 -0.376334
H -0.471939 -3.098301 0.201414
H -3.451253 -1.557466 -0.997290
H -2.924608 -2.932460 0.022575
H -3.561342 -1.428108 0.752166
H -0.277084 4.013656 0.586951
H -1.750252 3.232343 -0.094723
H -1.090705 2.694656 1.483807
H 4.111393 0.887838 -0.622728
H 3.855650 -0.639330 0.298412
H 3.215200 -0.475191 -1.365983

CoQ0-, A
E = -649.797118
C -1.422089 0.395982 -0.187537
C -1.697403 -1.026645 -0.037840
C -0.672466 -1.924837 0.156273
C 0.720936 -1.548115 0.252454
C 0.996495 -0.120107 0.107250
C -0.019788 0.795079 -0.099184
C -3.137243 -1.462189 -0.123769
O 1.641314 -2.396528 0.465397
O 2.288876 0.343780 0.261032
C 3.217991 -0.122972 -0.716318
O 0.314517 2.120535 -0.305831
C -0.189668 3.028955 0.671829
O -2.349382 1.238090 -0.398487
H -0.881523 -2.992364 0.264353
H -3.580584 -1.174019 -1.089650
H -3.224052 -2.551142 -0.001807
H -3.747985 -0.966456 0.646951
H 0.136865 4.029785 0.357948
H -1.285737 2.986420 0.715619
H 0.241194 2.804141 1.662981
H 4.193647 0.298064 -0.437704
H 3.265786 -1.219595 -0.716197
H 2.939187 0.247236 -1.718320

CoQ0, C
E = -649.720120
C -0.152152 0.846365 -0.110021
C 1.351707 0.884022 -0.000683
C 2.111448 -0.405201 0.061720
C 1.427464 -1.562929 0.018608
C -0.045952 -1.621284 -0.128479
C -0.814689 -0.349553 -0.161933
O 1.939265 1.952098 0.052784
C 3.602811 -0.307550 0.176252
O -0.607316 -2.708456 -0.245873
O -2.157177 -0.405940 -0.404131
C -2.976291 -1.103883 0.555054
O -0.657165 2.086406 -0.174076
C -2.050531 2.353423 0.054293
H 1.922214 -2.533934 0.064970
H 3.885544 0.259262 1.074357
H 4.055913 -1.304599 0.219607
H 4.020153 0.243339 -0.678278
H -2.112031 3.443482 0.128186
H -2.666535 1.989029 -0.773346
H -2.380300 1.898091 0.997457
H -4.005157 -0.986640 0.199727

H -2.709728 -2.163832 0.601564
H -2.873820 -0.637531 1.547258

CoQ0-, C
E = -649.796516
C 0.019720 0.782894 -0.263848
C 1.430679 0.429077 -0.125666
C 1.724732 -0.982250 0.081015
C 0.713608 -1.915816 0.095825
C -0.684069 -1.591357 -0.087195
C -0.984538 -0.169312 -0.245025
O 2.352921 1.301290 -0.181440
C 3.171739 -1.366155 0.250727
O -1.588840 -2.482554 -0.110064
O -2.283920 0.237414 -0.478329
C -3.221754 -0.091605 0.545409
O -0.325746 2.097476 -0.515320
C 0.036802 3.029227 0.501870
H 0.937924 -2.977079 0.231395
H 3.616243 -0.863785 1.124337
H 3.275651 -2.453376 0.374682
H 3.769533 -1.045247 -0.616346
H -0.297261 4.013550 0.146581
H -0.486359 2.791775 1.445477
H 1.122107 3.031332 0.664097
H -4.193338 0.288289 0.201309
H -3.268562 -1.176729 0.700854
H -2.952774 0.414041 1.490162

CoQ0, B
E = -649.720197
C -0.390345 0.562237 -0.160039
C 1.053910 0.907960 -0.140585
C 2.075603 -0.180126 0.033993
C 1.663462 -1.459325 0.102787
C 0.237313 -1.839414 0.048978
C -0.792377 -0.743565 -0.081654
O 1.424133 2.069481 -0.287876
C 3.511674 0.243880 0.095800
O -0.104814 -3.009511 0.124636
O -2.030301 -1.248018 -0.131687
C -3.201193 -0.426309 0.008790
O -1.283132 1.565480 -0.418966
C -1.372543 2.634201 0.542763
H 2.359248 -2.292283 0.209071
H 3.787957 0.801251 -0.809918
H 3.676627 0.929366 0.939150
H 4.170534 -0.625372 0.203263
H -2.167828 3.291607 0.176493
H -1.652066 2.233018 1.529443
H -0.429155 3.184847 0.608085
H -4.028106 -1.139502 0.078233
H -3.145943 0.169892 0.928886
H -3.331379 0.230483 -0.856558

CoQ0-, B
E = -649.796517
C -0.019806 -0.782909 -0.263778
C -1.430705 -0.428910 -0.125597
C -1.724594 0.982464 0.081065
C -0.713356 1.915901 0.095845
C 0.684285 1.591261 -0.087198
C 0.984572 0.169180 -0.244914
O -2.353070 -1.300983 -0.181413
C -3.171561 1.366552 0.250675
O 1.589143 2.482371 -0.110162
O 2.283933 -0.237750 -0.478247
C 3.221877 0.091358 0.545386
O 0.325495 -2.097509 -0.515263
C -0.037409 -3.029352 0.501729
H -0.937553 2.977188 0.231425
H -3.768973 1.047823 -0.617493
H -3.616786 0.862403 1.122863
H -3.275135 2.453554 0.376818
H 0.297580 -4.013505 0.146846
H 0.484727 -2.791490 1.445793
H -1.122849 -3.032015 0.663071
H 4.193518 -0.288121 0.200987
H 3.268380 1.176465 0.701025
H 2.953265 -0.414612 1.490066

[CoQ0-2CH3]-
E = -570.062336
C 1.458568 0.294251 -0.000400
C 0.349419 1.269178 -0.000325
C -1.062778 0.755236 0.000181
C -1.330238 -0.732116 -0.000169
C -0.310967 -1.612601 -0.000284
C 1.114863 -1.194820 0.000009
O 0.549953 2.505909 -0.000242
O -2.038978 1.513328 0.000743
C -2.771379 -1.154710 -0.000241
O 1.987862 -2.060303 0.000561
O 2.656327 0.626551 0.000027
H -0.480740 -2.692450 -0.000391
H -3.292380 -0.744993 -0.877722
H -2.860317 -2.249157 0.000391
H -3.292813 -0.743785 0.876389

[CoQ0-2CH3]-
E = -569.974471
C 0.652420 -0.044723 -0.232194
C -0.485158 -0.885515 -0.170152
C -1.900979 -0.195047 0.070600
C -1.866468 1.270582 0.177262
C -0.763779 2.004007 0.114580
C 0.614324 1.369418 -0.079746
O -0.496102 -2.123959 -0.273621
O -2.903520 -0.879712 0.148928
O 1.591215 2.145669 -0.091731
O 1.889945 -0.624263 -0.503218
C 2.455658 -1.311695 0.605093
H -0.744918 3.093495 0.198371
H 3.414960 -1.727106 0.264317
H 2.640983 -0.618618 1.444167
H 1.800562 -2.131810 0.937625

[CoQ0-2CH3]-
E = -569.972131
C -0.451909 -0.882914 -0.168143
C 0.671232 -0.011343 -0.246306
C 0.620964 1.410958 -0.084231
C -0.770345 1.925766 0.103968
C -1.915422 1.261365 0.181160
C -1.849275 -0.233481 0.058758
O 1.917344 -0.570067 -0.510455
C 2.516411 -1.197052 0.617243
O 1.593944 2.192728 -0.083647
O -2.865722 -0.904198 0.128480
O -0.406743 -2.126030 -0.257564
H -2.903400 1.705955 0.318751
H 3.478299 -1.606932 0.277269
H 2.701250 -0.463312 1.420588
H 1.883341 -2.014977 0.994187

[CoQ0-CH3]
E = -609.831333
C 0.043218 1.254978 -0.000030

C 1.507019 0.748588 0.000026
C 1.768674 -0.721069 -0.000035
C 0.722007 -1.576086 -0.000057
C -0.708898 -1.186378 0.000029
C -1.022233 0.249040 -0.000039
O 2.416340 1.553989 0.000146
C 3.201161 -1.160440 -0.000074
O -1.581646 -2.061381 0.000143
O -2.242649 0.755968 -0.000078
C -3.452752 -0.039882 -0.000006
O -0.180563 2.457255 -0.000022
H 0.877283 -2.656457 -0.000101
H 3.724955 -0.757528 0.878318
H 3.279179 -2.253538 0.000029
H 3.724852 -0.757718 -0.878618
H -4.254416 0.703521 -0.000053
H -3.496450 -0.668757 -0.894223
H -3.496421 -0.668685 0.894253

[CoQ0-CH3]-
E = -609.935508
C -0.271793 1.070159 -0.183417
C 1.262577 0.907275 -0.012220
C 1.855453 -0.463202 0.068396
C 1.022160 -1.518269 0.013870
C -0.470986 -1.425736 -0.117113
C -1.038616 -0.123109 -0.231611
O 1.991861 1.889816 0.048910
C 3.349342 -0.572674 0.206975
O -1.106716 -2.507646 -0.117869
O -2.410433 -0.035361 -0.445928
C -3.138405 0.451867 0.673557
O -0.725079 2.232649 -0.256509
H 1.396789 -2.544341 0.070339
H 3.699888 -0.056575 1.113495
H 3.658476 -1.625794 0.252388
H 3.859975 -0.085924 -0.637612
H -4.195684 0.486511 0.373074
H -3.032349 -0.229550 1.536279
H -2.802545 1.462131 0.952586

[CoQ0-CH3]-
E = -609.936925
C 0.761803 0.390909 -0.240180
C -0.441045 1.145767 -0.124139
C -1.743424 0.378713 0.039024
C -1.759895 -0.966833 0.120014
C -0.519728 -1.765292 0.042478
C 0.828890 -1.026541 -0.159866
O -0.515557 2.397708 -0.148486
C -2.993527 1.207033 0.111777
O -0.563534 -2.987899 0.128733
O 1.871342 -1.710048 -0.229000
O 1.933486 1.100890 -0.486240
C 2.809156 1.170978 0.630955
H -2.687363 -1.531818 0.245283
H -3.102323 1.815801 -0.797489
H -3.880478 0.571480 0.237622
H -2.930447 1.923936 0.942819
H 3.691462 1.741922 0.306964
H 2.328846 1.699949 1.473110
H 3.121032 0.165132 0.951248

[CoQ0-CH3]-
E = -609.835076
C 0.694142 -0.243905 0.167111
C 1.267440 1.102262 -0.242801
C 0.539754 2.279645 0.158856
C -0.816711 2.190652 0.170533
C -1.521431 0.894669 0.157628
C -0.664067 -0.334079 0.197229
O 2.251225 1.082604 -1.010113
O -2.750669 0.785260 0.300683
O -1.274351 -1.562074 0.347966
C -2.060104 -1.986567 -0.765243
O 1.439021 -1.387049 0.149506
C 2.807599 -1.276466 0.553307
H -1.467984 3.032211 0.440295
H 3.131478 -2.300331 0.780355
H 3.417172 -0.838564 -0.244966
H 2.898476 -0.657080 1.458386
H -2.480551 -2.962627 -0.489432
H -2.872327 -1.276015 -0.967050
H -1.427793 -2.104790 -1.661652

OH
E = -75.740612
O 0.000000 0.000000 0.108808
H 0.000000 0.000000 -0.870466

OH-
E = -75.808423
O 0.000000 0.000000 0.107740
H 0.000000 0.000000 -0.861922

O-
E = -75.138730
O 0.000000 0.000000 0.000000

[CoQ0-H]-
E = -649.128929
C -0.044555 0.803131 -0.181486
C -1.459020 0.326195 -0.053413
C -1.681528 -1.140864 -0.024396
C -0.623408 -1.995166 -0.014574
C 0.675529 -1.473753 0.308623
C 0.999330 -0.069016 -0.168773
O -2.383900 1.148049 -0.165861
C -3.106644 -1.586968 -0.280578
O 1.512006 -2.015762 1.061096
O 2.275130 0.410651 -0.223897
C 3.295151 -0.505883 -0.634461
O 0.175259 2.148993 -0.393188
C -0.113245 2.988265 0.724016
H -3.538130 -1.091625 -1.166167
H -3.117446 -2.674788 -0.423508
H -3.768141 -1.333941 0.564780
H 0.101162 4.015450 0.400645
H -1.168390 2.904907 1.016472
H 0.537870 2.733525 1.577420
H 4.155708 0.116251 -0.911852
H 3.551775 -1.194831 0.177823
H 2.967972 -1.086036 -1.510558

[CoQ0-H]-
E = -649.179827
C -0.227287 0.793260 -0.166876
C -1.556681 0.187758 -0.183517
C -1.620164 -1.316495 -0.016575
C -0.396931 -2.046129 0.136989
C 0.873376 -1.466662 0.164913
C 0.911965 0.035121 -0.036693
O -2.571079 0.885692 -0.337640
C -2.866412 -1.881520 -0.013243
O 1.973909 -2.072000 0.366612
O 2.077729 0.723062 -0.031588
C 3.336458 0.109103 -0.348544
O -0.121107 2.155953 -0.395125

```
C -0.562727 2.968386 0.688872
H -3.752641 -1.261886 -0.130853
H -2.991098 -2.958843 0.105992
H -0.424009 4.011354 0.372787
H -1.624162 2.787680 0.909434
H 0.046092 2.779982 1.590003
H 3.992271 0.957234 -0.593276
H 3.727086 -0.463734 0.497670
H 3.248095 -0.563708 -1.208850
H -0.446820 -3.126675 0.287069
```

```
[CoQ0-H]-
E = -649.153226
C    0.060274    0.811216   -0.094211
C    1.439330    0.341016    0.022662
C    1.618450   -1.104354    0.056760
C    0.539804   -1.949967   -0.026121
C   -0.833378   -1.503081   -0.167310
C   -1.018461   -0.067481   -0.183709
O    2.434540    1.125956    0.099457
C    3.030894   -1.614020    0.184979
O   -1.797703   -2.324588   -0.273318
O   -2.279501    0.466291   -0.378665
C   -3.227736    0.151770    0.640437
O   -0.272806    2.143556   -0.131718
C    0.582247    3.189804   -0.050518
H    3.504184   -1.247521    1.109251
H    3.661103   -1.250144   -0.641259
H    0.069309    4.142616   -0.146364
H    1.649104    3.017518   -0.002068
H   -4.173955    0.618086    0.333300
H   -3.352076   -0.934979    0.733520
H   -2.911250    0.581836    1.607129
H    0.681545   -3.033758   -0.005916
H    3.047251   -2.712806    0.188542
```

```
[CoQ0-H]-
E = -649.154002
C  -0.068869    0.804396   -0.188148
C  -1.437233    0.337292   -0.156900
C  -1.620981   -1.102225    0.019661
C  -0.540679   -1.944692    0.118578
C   0.835750   -1.503559    0.072994
C   1.019599   -0.061331   -0.080842
O  -2.432443    1.119682   -0.278979
C  -3.037195   -1.611148    0.065298
O   1.801233   -2.322138    0.169631
O   2.252702    0.537980   -0.134159
C   3.449144   -0.088357   -0.055831
O   0.189420    2.145327   -0.413509
C  -0.290803    3.025781    0.601277
H  -3.581819   -1.335892   -0.850836
H  -3.594718   -1.149143    0.894763
H  -0.030291    4.041884    0.274489
H   0.211311    2.816548    1.562301
H   4.279902    0.603806   -0.158727
H   3.492629   -1.166818    0.020853
H  -0.679613   -3.021763    0.238986
H  -3.058393   -2.703689    0.182691
H  -1.378690    2.931309    0.715106
```
